# Differentiation and comparison of *Wolfiporia cocos* raw materials based on multi-spectral information fusion and chemometric methods

**DOI:** 10.1038/s41598-018-31264-1

**Published:** 2018-08-29

**Authors:** Yan Li, Yuanzhong Wang

**Affiliations:** Institute of Medicinal Plants, Yunnan Academy of Agricultural Sciences, Kunming, 650200 Yunnan China

## Abstract

In order to achieve the target of deeper insight into the differentiation and comparison of *Wolfiporia cocos*, a total of 350 samples including distinct growth patterns, various collection regions and different medicinal parts were investigated using multi-spectral information fusion based on ultraviolet (UV) and Fourier transform infrared (FT-IR) spectroscopies coupled with chemometrics. From the results, the discrimination of samples was obtained successfully and good classification performances were shown according to partial least squares discriminant analysis (PLS-DA) models. Comparatively, the distinctness of chemical information in the two medicinal parts of *W. cocos* were much more than that in the same part with different growth patterns and collection areas. Meanwhile, an interesting finding suggested that growth patterns rather than geographical origins could be the dominant factor to effect the chemical properties of the same part samples, especially for the epidermis. Compared with the epidermis samples, there were better quality consistency for the inner part of *W. cocos*. Totally, this study demonstrated that the developed method proved to be reliable to perform comparative analysis of *W. cocos*. Moreover, it could provide more comprehensive chemical evidence for the critical supplement of quality assessment on the raw materials of *W. cocos*.

## Introduction

Edible and medicinal mushrooms are widely known as natural resources with valuable health benefits and have traditionally played important roles in food, agriculture and pharmaceuticals all around the world^1,2^. These mushrooms are appreciated not only for the taste and flavor but also for the nutritional properties and often sold in the local markets to constitute an element of human diet since they are rich in dietary protein, carbohydrates, essential amino acids, vitamins as well as minerals^3,4^. Many species of mushrooms consumed in the daily life have always been collected from wild habitats on the basis of ethnomycological knowledge and some efforts have been made to cultivate the mushrooms artificially on a commercial scale (Supplementary Figs S1 and S2). With the increasing awareness on the demand of edible and medicinal mushrooms, the natural raw materials are in short supply and the cultivated species have become the major substitutes of the wild ones although these species have different growing conditions. Hence, due to the diversification of mushroom raw materials, the quality of them has become a very important issue of public concern. The investigation of differences among the mushrooms with unprocessed states are of essential importance for the quality assurance and estimation of edible and medicinal values before they are converted to the final products.

However, edible and medicinal mushrooms are complicated systems and their multi-component characters hamper identification of their bioactive constituents^5,6^. In addition, the special qualities of these mushrooms are associated with not only one or several components, but also the synergistic effect of the holistic composition^7^. Thus, the comprehensive quality comparison and evaluation workflows have been developed with the aid of modern analytical techniques. In general, spectroscopic instrumental technique is rapid, simple and cost-effective, and it is one of the most commonly used approaches for the researches of complicated biological materials^8,9^. With the advances of spectral technologies, multi-spectral information fusion methods which depend on the combination of the information from different spectroscopic measurements and performed with improved computer capacity and powerful chemometric tools have facilitated to extend in an increasing number of fields including food, plants, drugs etc.^10–12^. For example, Dankowska *et al*.^13^ demonstrated that fluorescence combined with ultraviolet and visible (UV-Vis) spectroscopies could give a complementary effect for the quantification of roasted *Coffea canephora var. robusta* and *Coffea arabica* concentration in blends. What’s more, near-infrared (NIR) and mid-infrared (MIR) spectroscopies could be fused together for identifying rhubarb samples collected from different regions of China and the improved classification model could also be presented^14^. For edible mushrooms, Yao *et al*.^15^ established a quality evaluation strategy based on UV and MIR spectral techniques for the characterization of *Leccinum rugosiceps* with different geographical origins. Thereby, the integration of different spectroscopic methods could allow more efficient management of spectra and chemical information obtained from the tested samples owing to increase the global investigation ability and decrease the uncertainty of each individual result, which may have strong potential to explore the differences and quality of raw materials of edible and medicinal mushrooms.

*Wolfiporia cocos* (F.A. Wolf) Ryvarden & Gilb. is a renowned saprophytic mushroom belonging to Polyporaceae. This species is an important sub-material for the well-known snack called “Tuckahoe Pie” (Supplementary Fig. S3) and also used as one of the most common materials in traditional medicines of China and some other Asian countries^16,17^. The inner parts and epidermis are the medicinal parts of *W. cocos* that officially documented in Chinese Pharmacopoeia for a long time^18^. Besides, *W. cocos* is considered to have anti-tumor, anti-oxidant, anti-rejection and anti-hyperglycemic activities according to the major active components, and this species often used as a diuretic, sedative as well as tonic for treating various symptoms including gastritis, gastric atony, acute gastroenteric catarrh, dizziness, nausea and emesis^19–21^. As an important edible and medicinal mushroom, the raw materials of *W. cocos* are widely distributed in Yunnan, Guizhou, Sichuan, Hubei, Anhui, Guangxi and Fujian provinces in China, and the typical natural habitat is Yunnan Province. As a matter of fact, wild *W. cocos* is rare in nature and thus has always been highly cherished, and the cultivated species has been commercially in demand (Supplementary Fig. S4). Because of their diverse growing conditions, the wild-grown and cultivated *W. cocos* resources may contain different levels of the quality and medicinal efficacy. Therefore, the differentiation and comparison of *W. cocos* raw materials should be pointed out firstly and it is related to the recognition of certain quality characteristics of the final certified mushroom products.

In this study, to gain further insights into the similarities and differences among the wild as well as cultivated *W. cocos* raw materials with various collection regions, we investigated the samples by multi-spectral information fusion based on UV and Fourier transform infrared (FT-IR) spectroscopies combined with chemometric methods in terms of partial least squares discriminant analysis (PLS-DA) and hierarchical cluster analysis (HCA). Three factors including growth patterns, geographical origins and different medicinal parts were considered and the influence of these factors to *W. cocos* was also compared. The results may improve the current knowledge and pave the way for future exploitation and utilization of this species.

## Results and Discussion

### UV spectra characterization

**Figure 1:**
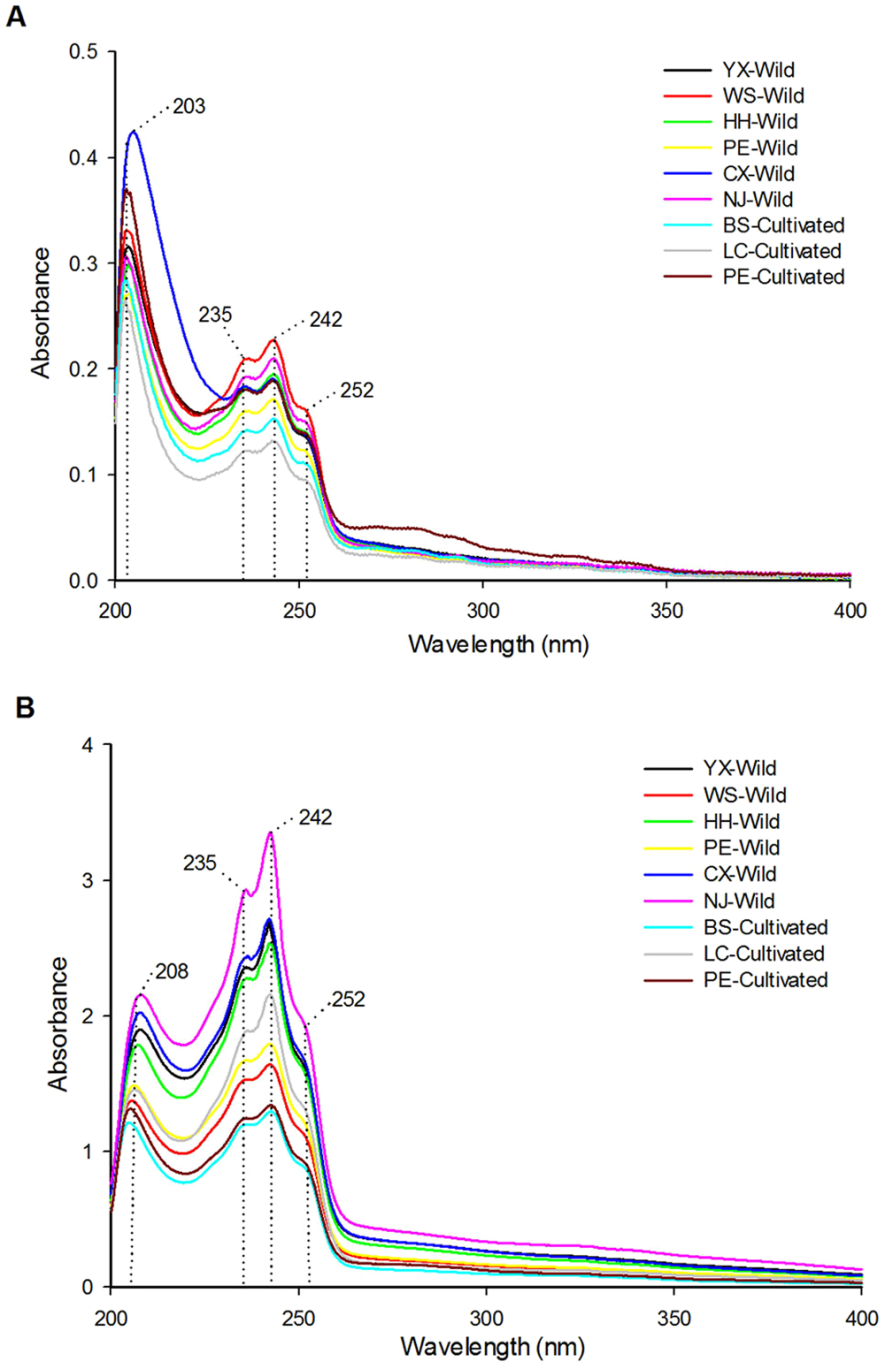
Average original UV spectra of the wild and cultivated inner part (**A**) and epidermis (**B**) of *W. cocos* samples with different collection regions.

Fig. 1 presents stacked UV absorption spectra in the range of 200–400 nm recorded for different parts of wild and cultivated *W. cocos* samples originated from eight regions. By carefully examination of the UV spectra corresponding to the inner parts (Fig. 1A), it is worth noting that the spectral profiles appear similar shape with each other on the basis of visual inspection, suggesting a homogeneous chemical composition among the inner part samples, despite their growth patterns and collection regions were different. Obviously, the characteristic region of the spectra is found from 200 to 270 nm with the strong absorption bands at around 203, 235 and 242 nm and a shoulder peak at about 252 nm. Especially, the maximum absorption of each spectrum is around 203 nm. The inner part of *W. cocos* is a complex mixture system, which usually contains many triterpenoids and polysaccharides^19^. Based on the previous studies, the absorption bands mentioned above may be associated with the existence of some triterpenoids^22,23^. What’s more, for the average spectra, they present some differences in the absorption intensities. In detail, the spectra of wild samples are somewhat similar and have relative high absorbance than that of the cultivated species except the cultivated samples collected from Pu’er region. In addition, whether the wild or the cultivated samples, the peak heights of spectra vary from samples to samples in terms of the geographical origins. Generally, when the chemical component was in high concentration, the corresponding absorption intensity was high, too. It indicated that the contents of the chemical components in the inner part samples may be a bit different based on the various growth patterns and collection regions.

On the other hand, as can be seen in the average raw UV absorbance spectra relative to epidermis samples (Fig. 1B), the shape of the mean exhibits a clear similarity and the presence of informative features of the spectra is mainly found in the wavelengths of 208, 235, 242 and 252 nm, which could be explained by the existence of some triterpenoids in the epidermis of *W. cocos*^24,25^. Especially, the strongest absorption appears at about 242 nm, probably indicating the presence of a heteroannular diene moiety of dehydropachymic acid^26^. In addition, it also shows obvious difference in the absorbance and the cultivated epidermis samples have lower peak heights than those of wild ones apart from the cultivated samples obtained from Lincang region, and the epidermis of *W. cocos* from different geographical regions display the changes of UV absorption intensities. It was similar to the inner parts that growth patterns and collection regions could lead to the distinctness among the contents of chemical components in epidermis of *W. cocos*. Furthermore, although the UV spectra of the two medicinal parts samples have three common characteristic absorption peaks with the wavelengths of 235, 242 and 252 nm, the absorbance of the epidermis is dramatically higher than that of the inner parts, which also indicated the difference of the content of chemical components. Consequently, it suggested that the content of chemical components in *W. cocos* could be influenced by growth patterns, geographical regions and different medicinal parts.

### Examination of visible and original FTIR spectra

**Figure 2:**
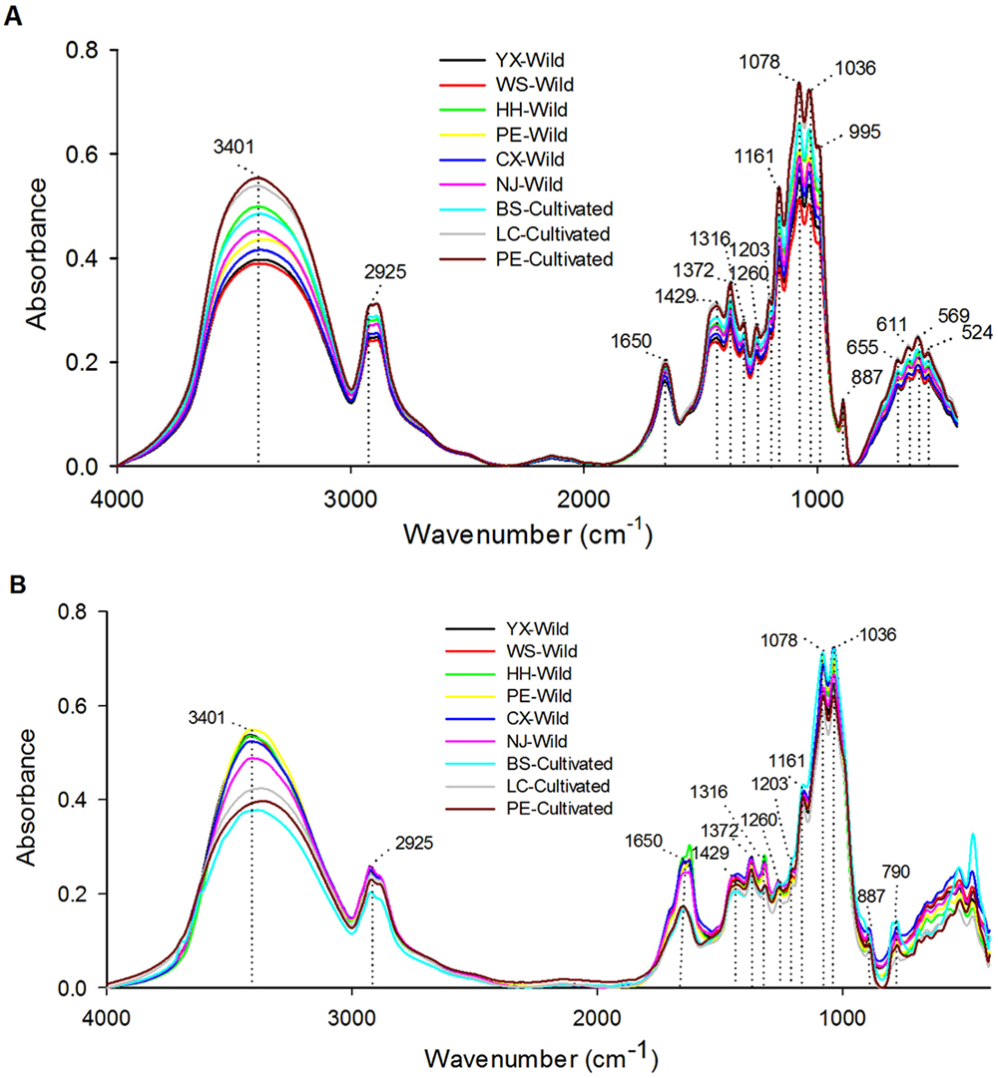
Average raw FTIR spectra profiles of the wild and cultivated inner part (**A**) and epidermis (**B**) of *W. cocos* samples collected from different regions.

Average original FTIR spectra of the wild and cultivated inner part and epidermis samples with different collection regions are shown in Fig. 2 and a number of common characteristic absorption peaks are observed. From the overall view of the spectra recorded for inner parts (Fig. 2A), it shows a typical major broad stretching peak at 3401 cm^−1^ for the -OH of polysaccharide, triterpene and sterol, which have been known as the most biologically active substances in *W. cocos*^19^. The common backbone at 2925 cm^−1^ corresponds to the C-H stretching vibration of -CH_2_-. What’s more, although unambiguous identification of the molecular source of unique chemical information characteristics in mid-infrared spectra of biological material is relative difficult, following peaks in the region of 1800–400 cm^−1^ which contains a large number of bands and is rich in structural information could present spectral informative features in various compounds. The strong absorption peaks around 1650 cm^−1^ are mainly attributed to C=O and C-N stretching vibrations as well as bending vibration of N-H groups, confirming the presence of amide I band of protein components^27^. Absorbance at approximately 1372 cm^−1^ may due to the presence of triterpene compounds (CH_2_=CH-CH_3_) while peaks at 1260 and 1203 cm^−1^ belong to the amide III band and C-C stretching band, respectively^28^. Twin peaks with higher absorbance which appear at 1078 and 1036 cm^−1^ are likely to suggest the structures in chitin, a major structural polysaccharide in mushrooms^29^. In addition, the region between 900 and 400 cm^−1^ is mainly identified as the content of carbohydrate^30–32^. Moreover, the small but obvious peak around 887 cm^−1^ may indicate the existence of C=CH_2_ presented in triterpene compounds^33^. Totally, 17 common absorption peaks of the FTIR spectra of inner part samples have been presented and no significant visible differences among the spectral shapes and peak positions as well as a few diversities of the absorption intensities could be observed, suggesting the relative similar chemical components existed in the inner part of *W. cocos* even they were obtained from two growth patterns and eight geographical regions. In other words, the growth patterns and collection regions may have few impacts on the chemical composition of inner part of *W. cocos*.

On the contrary, for the FTIR spectra of the epidermis samples (Fig. 2B), despite there are 13 obvious common absorption peaks at 3401, 2925, 1650, 1429, 1372, 1316, 1260, 1203, 1161, 1078, 1036, 887 and 790 cm^−1^, which also appear in the spectra of the inner parts except the last one, several differences of the spectral shapes are highlighted in the lower part region of spectra, especially from 790 to 400 cm^−1^. In terms of the information of the *W. cocos* samples, it suggested that some chemical components in the epidermis samples may change with the growth patterns and geographical areas. Furthermore, both of the two parts samples have some common characteristic absorption peaks in the FTIR spectra, indicating the similar chemical constituents of them. It is similar to the previous summary that some triterpenes in *W. cocos* were isolated from both the inner part as well as the epidermis, including pachymic acid, dehydropachymic acid, 3-*epi*-dehydrotumulosic acid, dehydrotumulosic acid, dehydrotrametenolic acid, 3-*epi*-dehydrotrametenolic acid, poricoic acid A, poricoic acid B, 16-deoxyporicoic acid B, etc.^19^. In addition, the region of 790–400 cm^−1^ in the spectra also contains the main distinction of spectral features between the inner part and epidermis. It implied that apart from the growth patterns and collection areas, the chemical constituents in *W. cocos* could be affected by different medicinal parts which is similar to the initial analysis of UV spectra. Therefore, the growth patterns, geographical regions and different parts were likely to have influences on the chemical composition of *W. cocos*. What’s more, the first two factors have different effect degrees on the two medicinal parts of *W. cocos*.

### Differentiation of *W. cocos* with different growth patterns

An inability to assign all spectral characteristics is a general feature of spectra of complex biological materials, but the presence of such spectral details could indicate the detection of a significant number of information, which may be usefully interrogated by chemometric methods. In order to investigate whether the growth patterns, geographical regions and different parts could affect the chemical property of *W. cocos*, PLS-DA was used to establish the classification models based on the fused spectral information after selecting the key variables of UV and FTIR spectra according to CARS method.

**Figure 3:**
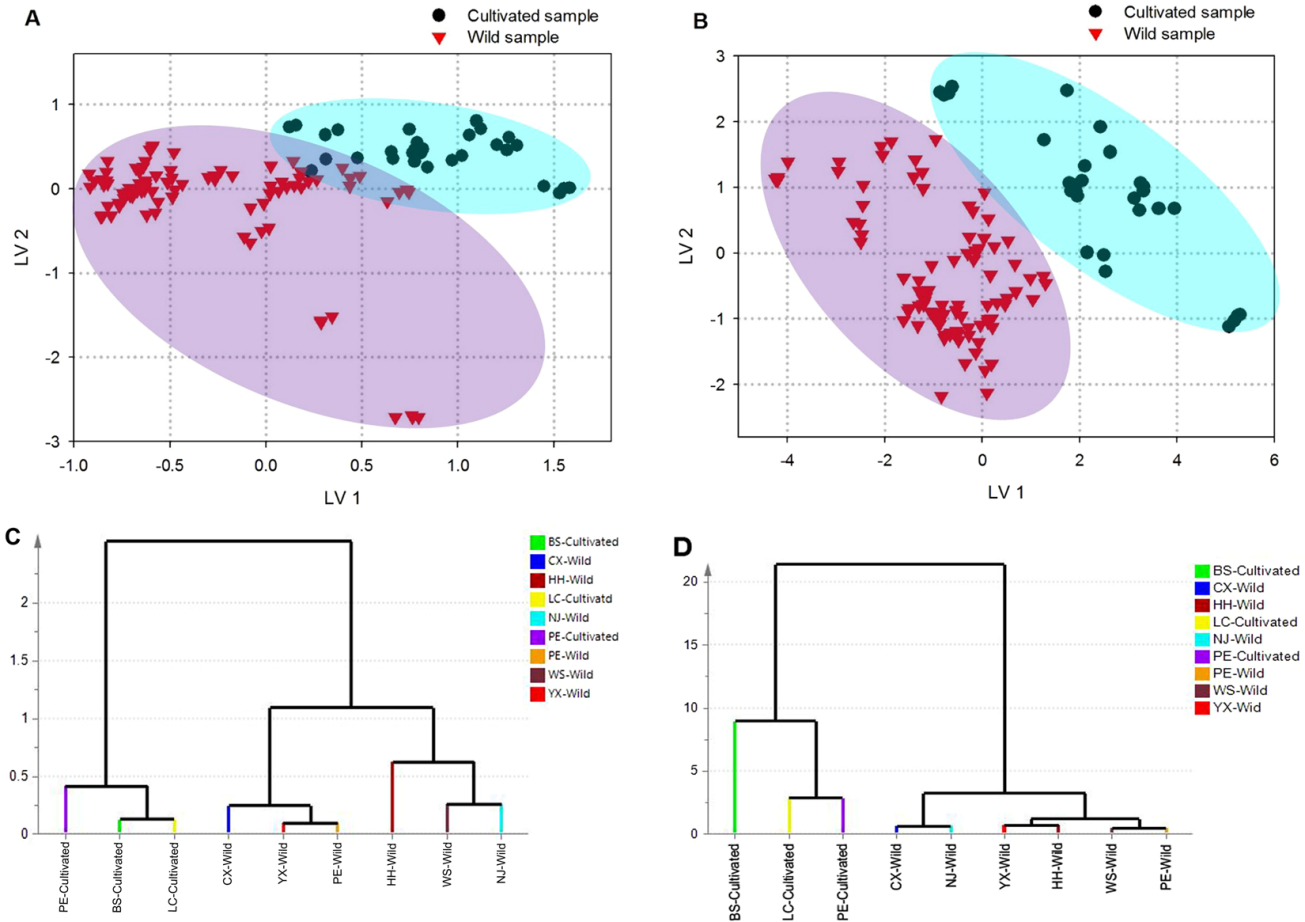
PLS-DA **s**core plots of the inner part (**A**) and epidermis (**B**) of *W. cocos* samples with different growth patterns; HCA dendrograms of the inner part (**C**) and epidermis (**D**) of *W. cocos* samples based on the key variables selected by CARS according to different growth patterns.

In regard to the comparison of the inner part samples with two different growth patterns, there were 121 and 176 variables respectively selected from the UV and FTIR spectra to form the fusion matrix. As a result, the first 10 LVs representing 97.8% of the total variance in the joint spectral information were chosen according to the lowest root mean square error of cross validation (RMSECV) to construct PLS-DA model. The goodness of fit of R^2^Y was 97.1%, and the goodness of prediction Q^2^Y was 94.2% which underlined the predictive ability of the model. Additionally, the response permutation test (Y scrambling) revealed no overfitting with R^2^ from 0.24 to 0.47, Q^2^ from −1.29 to −0.22, R^2^Y-intercept of 0.29 and Q^2^-intercept of −0.90 (Table 1). In this situation, samples in the calibration set are correctly classified and the accuracy of validation set is 98.08%, as shown in Table 1. It showed that almost all the inner part samples could be distinguished as their growth patterns. In addition, analyzing the average values of sensitivity and specificity observed, all values are more than 98.72%, suggesting good separation of the samples. It is noteworthy that the quality of the model can be also evaluated by efficiency and MCC with the average values more than 98.71% and 0.95, respectively, which demonstrated good classification performance of the model. Besides, more visual information is exhibited in the score plot of the first two LVs (Fig. 3A). This figure displays a good discrimination of the wild and cultivated inner part samples and the cultivated ones are more closely grouped in a cluster than those of the wild species, comparatively. In detail, both LV 1 and LV 2 are determined mainly by positive scores for the cultivated samples as well as the wild ones distribute in other three quadrants. It showed that the inner parts of *W. cocos* were as diverse in the growth patterns. Moreover, in order to obtain the meaningful information of the spectra responsible for the separation of samples, variable importance in projection (VIP) scores were used. The spectral information with VIP scores larger than 1 would be viewed as important variables for the developed PLS-DA model^34^. The VIP scores shown in Table S1 confirms that the wavelengths considered to be significantly contributive to the discrimination of growth patterns mainly distribute in the region of 204.5–253 nm which comprises approximately 62% of the UV spectral information while the wavenumbers responsible for the separation of classes are mainly included in the fingerprint region. Based on the previous studies, these spectral information mainly demonstrated the structures of triterpenes, polysaccharides and several protein components^22,23,27,29^. Hence, it seemed to imply that the differentiation of the inner part samples with two growth patterns may primarily due to the diversity of these constituents.

**Table 1:** Parameters of the PLS-DA models based on *W. cocos* samples with different growth patterns.

Parameters	Inner part			Epidermis		
	Wild samples	Cultivated samples	Average	Wild samples	Cultivated samples	Average
Calibration set						
Sensitivity (%)	100.00	100.00	100.00	100.00	100.00	100.00
Specificity (%)	100.00	100.00	100.00	100.00	100.00	100.00
Efficiency (%)	100.00	100.00	100.00	100.00	100.00	100.00
MCC	1.00	1.00	1.00	1.00	1.00	1.00
Accuracy (%)	100.00	100.00	100.00	100.00	100.00	100.00
Validation set						
Sensitivity (%)	97.44	100.00	98.72	100.00	100.00	100.00
Specificity (%)	100.00	97.44	98.72	100.00	100.00	100.00
Efficiency (%)	98.71	98.71	98.71	100.00	100.00	100.00
MCC	0.95	0.95	0.95	1.00	1.00	1.00
Accuracy (%)	98.08	98.08	98.08	100.00	100.00	100.00
RMSEE	0.07	0.07	0.07	0.07	0.07	0.07
RMSECV	0.11	0.11	0.11	0.14	0.14	0.14
Permutation test						
R^2^ (min-max)	0.24–0.47	0.24–0.47	—	0.13–0.43	0.13–0.43	—
Q^2^ (min-max)	−1.29–−0.22	−1.29–−0.22	—	−2.61–−1.00	−2.61–−1.00	—
R^2^-intercept	0.29	0.29	0.29	0.22	0.22	0.22
Q^2^-intercept	−0.90	−0.90	−0.90	−2.05	−2.05	−2.05

Meanwhile, the differentiation of epidermis of *W. cocos* with different growth patterns were also investigated. A total of 14 LVs were selected for the development of the classification model, representing 99.2% of the variance explained in the spectra data set. The R^2^Y and Q^2^ of this model were 97.7% and 93.4%, respectively while R^2^max and Q^2^max of permutation test were 0.43 and −1.00, separately, as well as R^2^-intercept and Q^2^-intercept were 0.22 and −2.05, respectively, suggesting good predictive capacity and no overfitting of this method. The parameters of the discrimination models for the calibration and validation set are shown in Table 1. It shows excellent performance for the studied samples with 100% sensitivity, specificity, efficiency and accuracy, and a MCC of 1. Thus, the PLS-DA model is useful for analyzing new samples of epidermis of *W. cocos* obtained by UV and FTIR spectroscopic data, predicting if they belong to wild species or cultivated ones. The evaluated parameters could give a general vision of the behavior of the model whereas the score plot could show more viewing information of the wild and cultivated epidermis samples. As can be seen from Fig. 3B, the LV1-LV2 score plot shows good clustering, and these two LVs are determined as good criteria for discriminating the epidermis samples with different growth patterns. Similar to the inner part samples, there were some differences of the chemical properties in the epidermis with these two growth patterns. The VIP scores are presented in Table S2 and there is a close agreement between the spectral discussion and the variables with greater importance. As can be seen in this table, the variables formatted in bold present distinguishable peaks of the samples, specifically the most obvious characteristic (242 nm) of the UV spectra and the main distinction of FTIR spectral features of the epidermis (474–410 cm^−1^), indicating the presence of some triterpenes and polysaccharides^26,31^. It could be inferred from the results that the differentiation of the wild and cultivated epidermis of *W. cocos* may be related to the diversity of triterpenes and polysaccharides.

In order to show the similarity between samples and verify the results of PLS-DA, we respectively divided the two part samples into nine classes based on the detailed information, particularly both the growth patterns and geographical origins were taken into consideration. Afterwards, the average fusion spectral information of each class was constructed by HCA. The dendrograms displayed in Fig. 3C,D provide very simple two dimensional plots of the data structure suggesting the merging objects and distances. It is clearly that all the classes are grouping into two main clusters relating to different growth patterns, not only the inner parts, but also the epidermis. One cluster consists the wild classes whereas the other one contains the cultivated classes, indicating the large similarity on the chemical constituents of samples with the same growth pattern as well as suggesting the obvious diversity between wild and cultivated samples, which were in direct agreement with the results obtained by PLS-DA. In short, on the basis of PLS-DA and HCA, it was concluded that growth patterns could affect the chemical property of *W. cocos*.

### Discrimination of *W. cocos* with different collection regions

**Figure 4:**
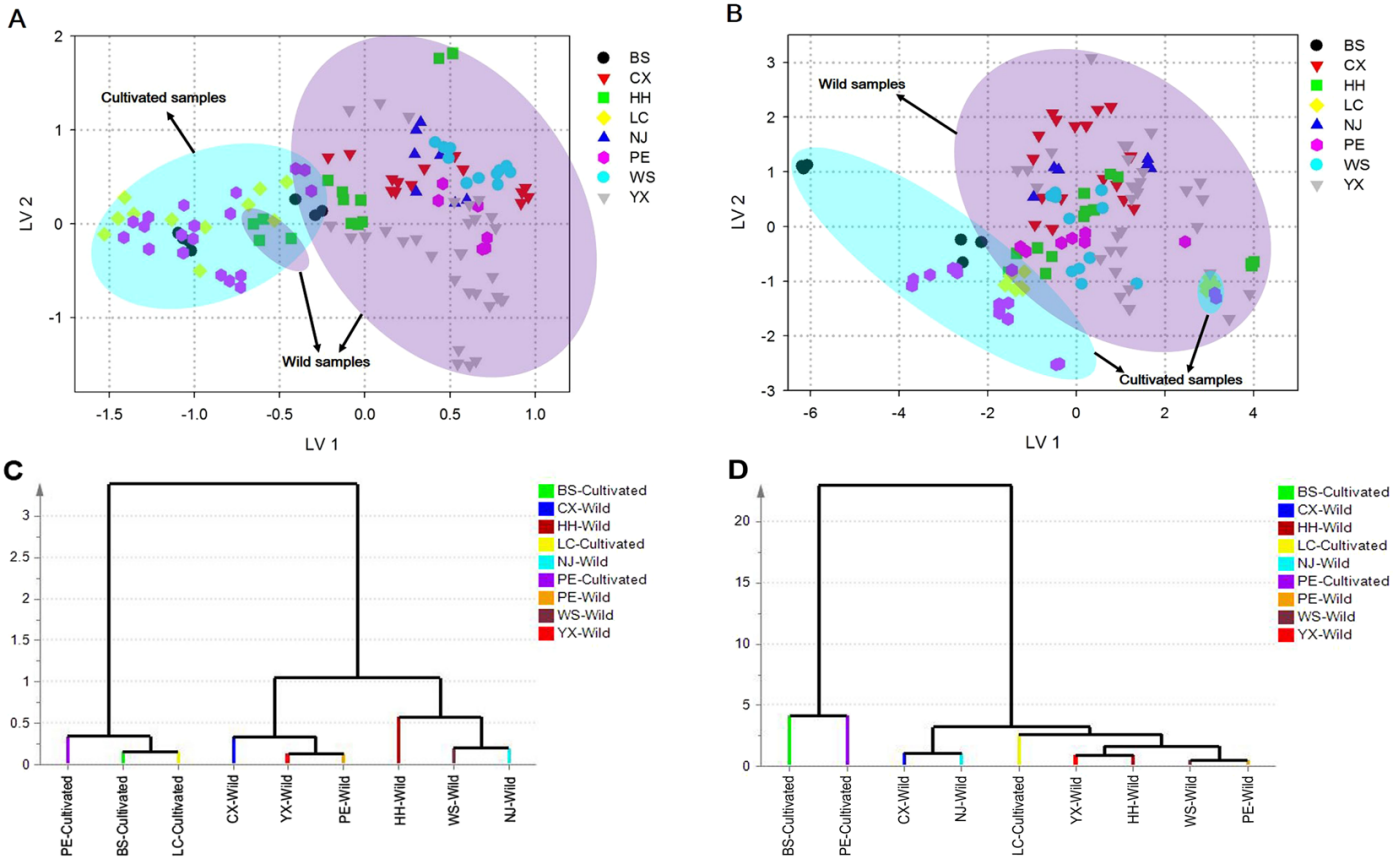
PLS-DA score plots based on first two LVs of the inner part (**A**) and epidermis (**B**) of *W. cocos* samples with different collection regions; HCA dendrograms of the inner part (**C**) and epidermis (**D**) of *W. cocos* samples after selecting the key variables by CARS in terms of different collection regions.

When studying on the distinctness among inner part samples obtained from different geographical regions, PLS-DA model was carried out to identify significant differences between the investigated groups of samples with 22 LVs which representing 99.6% of the total variance in the joint spectral information (R^2^Y = 80.9% and Q^2^ = 50.7%). Class permutation test also indicated that the model was rigorously built without overfitting (Table 2). The eight groups, BS, CX, HH, LC, NJ, PE, WS and YX, present comparably good discrimination results with 96.75% accuracy for calibration set and 94.23% accuracy for validation set as shown in Table 2. However, this model shows relative high sensitivity, specificity and efficiency for the calibration set with the average values of 97.80%, 96.77% and 97.27%, respectively. What’s more, MCC for the calibration set of each group ranges from 0.78 to 0.91 whereas that for the validation set is in the range of 0.55 to 0.86, which exhibits higher values in the former one as well. It clearly demonstrated that the inner parts of *W. cocos* originated from different geographical regions could be distinguished based on the integrated spectral information, which also suggested that the collection areas had influences on the chemical properties of the wild and cultivated inner parts. Moreover, from the score plot of LV 1 and LV 2 (Fig. 4A), it is interesting that the samples present a trend of separation that they are discerned into two major groups — one group consists of mostly wild samples whereas the other group contains the cultivated species even they were obtained from different regions. In other words, all the samples were firstly differentiated according to the growth patterns rather than the collection regions. Thus, considering the results of model parameters and the score plot, it could be inferred that the differences among the inner parts of *W. cocos* were prominent for the geographical origins as well as the growth patterns and the latter was regarded as the dominated one. In addition, a total of 85 variables from the fused spectral information are identified as important for the discrimination of the inner part samples collected from different regions (Table S3). In a word, the wavelengths at around 203 and 242 as well as the wavenumbers in the range of 1652 to 1022 cm^−1^ are responsible for the differentiation, which may suggest the existence of some triterpenes, proteins and polysaccharides^22,23,27,29^. On the whole, it was possible to estimate that the differences among the inner part samples with different collection regions may be related to the distinction of some chemical components such as triterpenes, proteins and polysaccharides.

**Table 2:** Classification parameters obtained for PLS-DA model of the inner part samples with different collection regions.

Parameters	Inner part								
	BS	CX	HH	LC	NJ	PE	WS	YX	Average
Calibration set									
Sensitivity (%)	100.00	100.00	92.86	100.00	100.00	96.00	100.00	93.55	97.80
Specificity (%)	96.55	96.23	97.25	96.43	96.55	96.94	96.43	97.83	96.77
Efficiency (%)	98.26	98.10	95.03	98.20	98.26	96.47	98.20	95.66	97.27
MCC	0.78	0.88	0.85	0.84	0.78	0.90	0.84	0.91	0.85
Accuracy (%)	96.75	96.75	96.75	96.75	96.75	96.75	96.75	96.75	96.75
Validation set									
Sensitivity (%)	100.00	100.00	100.00	80.00	66.67	100.00	100.00	92.86	92.44
Specificity (%)	93.88	93.18	93.48	95.74	95.92	92.86	93.75	94.74	94.19
Efficiency (%)	96.89	96.53	96.68	87.52	79.97	96.36	96.82	93.79	93.07
MCC	0.69	0.82	0.79	0.70	0.55	0.85	0.73	0.86	0.75
Accuracy (%)	94.23	94.23	94.23	94.23	94.23	94.23	94.23	94.23	94.23
RMSEE	0.11	0.15	0.17	0.14	0.12	0.16	0.17	0.21	0.15
RMSECV	0.16	0.24	0.25	0.20	0.20	0.26	0.23	0.32	0.23
Permutation test									
R^2^ (min-max)	0.16–0.45	0.17–0.47	0.14–0.47	0.18–0.47	0.13–0.45	0.19–0.47	0.17–0.45	0.22–0.49	—
Q^2^ (min-max)	−1.98–−0.35	−2.23–−0.49	−2.17–−0.49	−1.96–−0.32	−2.01–−0.27	−2.26–0.44	−2.24–−0.36	−2.37–−0.53	—
R^2^-intercept	0.33	0.35	0.36	0.34	0.33	0.38	0.35	0.39	0.35
Q^2^-intercept	−1.09	−1.27	−1.19	−1.10	−1.05	−1.42	−1.12	−1.44	−1.21

A similar summary of results obtained for the epidermis samples with eight collection regions is also shown in Table 3. In this case, PLS-DA model performance was produced based on the spectral information fusion using 144 UV spectral variables and 114 FTIR spectral variables even though it requires 29 LVs, much more than any other model. R^2^Y was calculated to be 89.9% and Q^2^ was 67.5%. All the R^2^ and Q^2^ values of the response permutation test were less than 0.5, and R^2^-intercept as well as Q^2^-intercept values presented goodness of fit of the established model (Table 3). Additionally, excellent performance is shown in the calibration set with the optimal values of the parameters. For the validation samples, the average sensitivity, specificity and efficiency are 91.24%, 90.43% and 90.62%, respectively, but the MCC values vary considerably over the range of 0.43–0.76. What’s more, high classification accuracies are obtained for all of the samples that the calibration set presents completely correct classifications while the validation set exhibits more than 90% of the samples are correctly classified. These results indicated that PLS-DA method was able to accurately and efficiently model the epidermis of *W. cocos* with different geographical origins, which also implied the collection regions could affect the chemical properties of the epidermis. With regard to the score plot based on the first two LVs (Fig. 4B), it is initial to found that most of the cultivated samples are discriminated with the wild species despite they were obtained from different geographical origins, which was almost identical to that of the inner part samples. It could be inferred that as compared to the collection regions, growth patterns played a more important role in the aspect of influencing the chemical components of epidermis of *W. cocos*. Moreover, in Table S4, the spectral information with the VIP scores greater than 1 that contributed significantly to the differences among epidermis samples obtained from various regions are formatted in bold, including the wavelengths of 208, 235 and 242 which also have obvious absorption in the UV spectra of epidermis samples and may be related to the existence of some triterpenoids of *W. cocos*^24,25^ as well as the functional groups of several polysaccharides, triterpenes and proteins such as the wavenumbers at around 1650, 1203, 1036, etc. of the FTIR spectra. Totally, the results implied that the diversity of epidermis samples with different geographical regions may be in connection with a number of triterpenes, polysaccharides and protein components, which was similar to that of the inner part of *W. cocos*.

**Table 3:** Classification parameters obtained for PLS-DA model of the epidermis samples with different collection regions.

Parameters	Epidermis								
	BS	CX	HH	LC	NJ	PE	WS	YX	Average
Calibration set									
Sensitivity (%)	100.00	100.00	100.00	100.00	100.00	100.00	100.00	100.00	100.00
Specificity (%)	100.00	100.00	100.00	100.00	100.00	100.00	100.00	100.00	100.00
Efficiency (%)	100.00	100.00	100.00	100.00	100.00	100.00	100.00	100.00	100.00
MCC	1.00	1.00	1.00	1.00	1.00	1.00	1.00	1.00	1.00
Accuracy (%)	100.00	100.00	100.00	100.00	100.00	100.00	100.00	100.00	100.00
Validation set									
Sensitivity (%)	100.00	87.50	100.00	100.00	66.67	90.00	100.00	85.71	91.24
Specificity (%)	89.80	90.91	89.13	89.58	91.84	90.48	89.58	92.11	90.43
Efficiency (%)	94.76	89.19	94.41	94.65	78.25	90.24	94.65	88.85	90.62
MCC	0.58	0.69	0.70	0.63	0.43	0.73	0.63	0.76	0.64
Accuracy (%)	90.38	90.38	90.38	90.38	90.38	90.38	90.38	90.38	90.38
RMSEE	0.07	0.11	0.13	0.12	0.09	0.15	0.11	0.15	0.12
RMSECV	0.11	0.20	0.22	0.17	0.16	0.29	0.22	0.28	0.20
Permutation test									
R^2^ (min-max)	0.16–0.44	0.19–0.44	0.20–0.43	0.19–0.45	0.16–0.45	0.16–0.44	0.19–0.44	0.26–0.44	—
Q^2^ (min-max)	−3.25–−0.48	−3.78–−0.82	−3.7–−0.75	−3.43–−0.57	−3.31–−0.65	−3.68–−0.81	−3.56–−0.72	−4.39–−0.87	—
R^2^-intercept	0.32	0.33	0.33	0.34	0.33	0.34	0.32	0.34	0.33
Q^2^-intercept	−1.79	−2.14	−2.01	−2.02	−1.69	−2.26	−1.86	−2.34	−2.01

Afterwards, HCA dendrograms of the inner part and epidermis of *W. cocos* are respectively presented in Fig. 4C,D. For the inner parts (Fig. 4C), all of the cultivated samples are together in one cluster at the left side in the HCA dendrogram while the wild species are clustered at the right side, which is roughly consistent with the results of the score plot acquired by PLS-DA. In addition, two subfractions can be recognized that samples collected from Chuxiong, Yuxi and Pu’er may have relatively closer relationships while the remaining wild samples may be similar with each other. Additionally, similar discrimination pattern is observed in the HCA dendrogram of the epidermis (Fig. 4D) that the cultivated samples are clustered together and discriminated from the wild ones except that from Lincang region, which is also similar to the results of PLS-DA. Nevertheless, for the wild species, although there are two subfractions as well, the epidermis samples display a difference in the order of the collection regions compared with the inner parts on account of samples with the geographical origins of Yuxi, Honghe, Wenshan and Pu’er could be grouped and the others are in one cluster. What’s more, growth pattern has much more influence on the epidermis samples than that of the collection regions because even the epidermis obtained from the same area, such as those from Pu’er, they are also separated into the wild and cultivated ones, respectively. Consequently, according to the results mentioned in this section, both growth patterns and collection regions could affect the chemical properties of not only the inner parts but also the epidermis and the former was a dominant one.

### Comparison of the two medicinal parts of *W. cocos*

**Figure 5:**
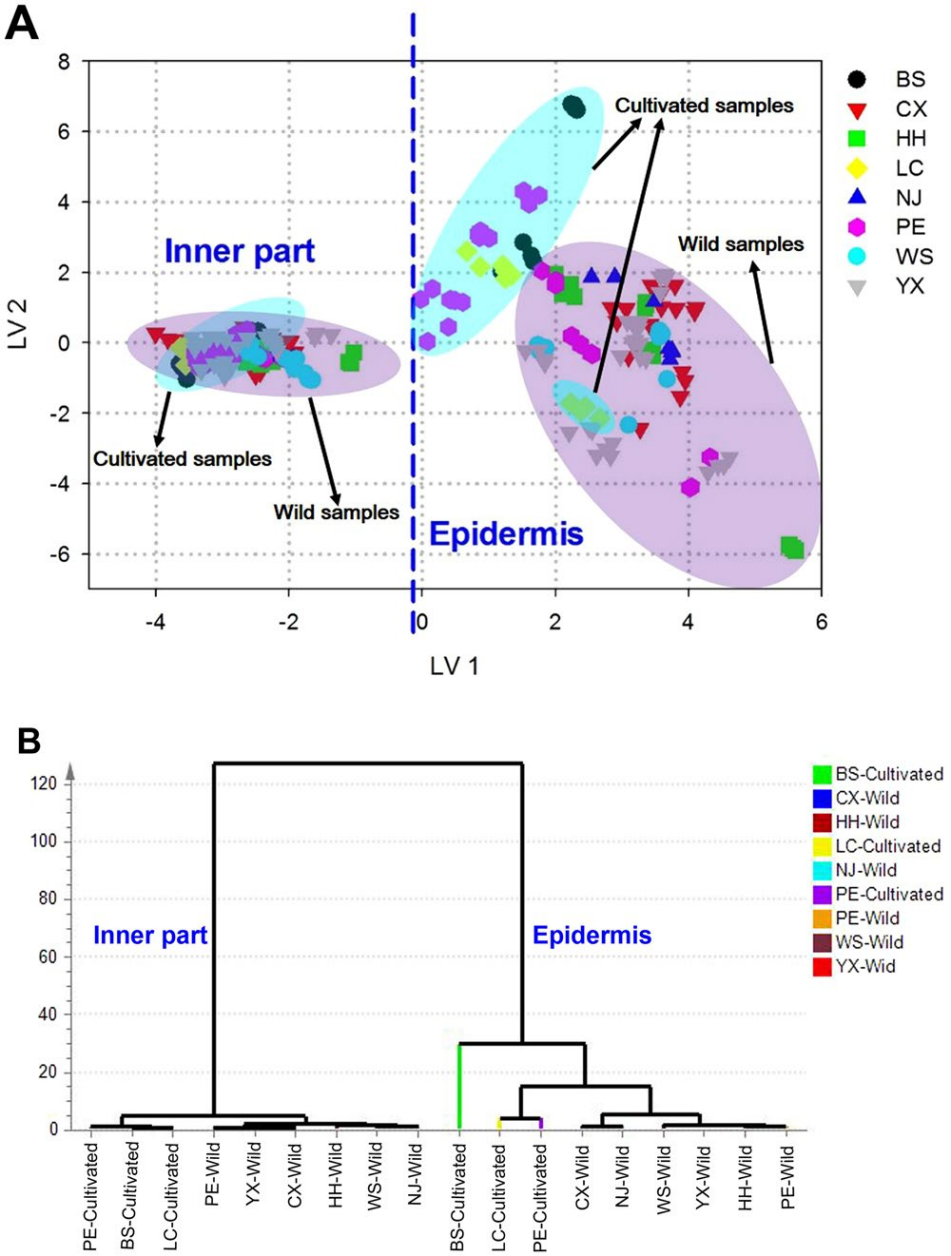
(**A**) PLS-DA score plot of different parts of *W. cocos*; (**B**) Dendrogram resulting of HCA after selecting the key variables by CARS according to different parts of *W. cocos*.

Apart from the growth patterns and collection regions, different medicinal parts may also have impacts on the chemical components of *W. cocos*. Similarly, after selecting the spectral information based on CARS method, the fusion matrix was carried out by PLS-DA for establishing the discrimination model of the two parts of *W. cocos*. Actually, the first 18 LVs accounting for 99.7% of the total variation were used and the detailed parameters are shown in Table 4. What’s more, this model achieved 99.3% goodness of fit (R^2^Y) with a goodness of prediction (Q^2^) of 98.6%. A response permutation test (Y scrambling) was used and showed no overfitting (R^2^ from 0.17 to 0.32, Q^2^ from −2.28 to −0.82, R^2^Y-intercept of 0.22 and Q^2^-intercept of −1.49, shown in Table 4). With respect to the calibration and validation sets of both the inner part and epidermis samples, all the values of sensitivity, specificity, efficiency and accuracy are 100%, respectively, indicating perfect performance of the classification model without any false prediction. In other words, the *W. cocos* samples could be exactly discriminated by the medicinal parts. After that, the two-dimensional plot using LV 1 and LV 2 is displayed in Fig. 5A. Firstly, individual samples belonging to the same part are grouped together and LV 1 plays a significant role in discerning them that all the inner part samples distribute in the left side while the positive scores on LV 1 are found for epidermis ones. What’s more, it is worthy of noticing that the inner part samples are closely distributed as well as the epidermis are relative scattered, suggesting the similarity of the quality consistency of the inner part of *W. cocos*. Then, in terms of further analysis, an interesting overall tendency is demonstrated that most of the epidermis samples are distributed relatively in accordance with their growth patterns rather than the geographical origins. The VIP scores in Table S5 present 13 UV spectral variables which mainly around the absorption peaks of 203, 235 and 252 nm that probably assigned to the presence of some triterpenoids of *W. cocos*^22–25^ and 62 FTIR spectral variables that mainly attributed to the existence of several proteins and polysaccharides^27,35^ are identified as most significant for explaining the differences of the two parts, indicating the diversity of the inner parts and epidermis of *W. cocos* may be related to these chemical components.

**Table 4:** Parameters of PLS-DA modeling on different parts of *W. cocos* samples.

Parameters	Inner part	Epidermis	Average
Calibration set			
Sensitivity (%)	100.00	100.00	100.00
Specificity (%)	100.00	100.00	100.00
Efficiency (%)	100.00	100.00	100.00
MCC	1.00	1.00	1.00
Accuracy (%)	100.00	100.00	100.00
Validation set			
Sensitivity (%)	100.00	100.00	100.00
Specificity (%)	100.00	100.00	100.00
Efficiency (%)	100.00	100.00	100.00
MCC	1.00	1.00	1.00
Accuracy (%)	100.00	100.00	100.00
RMSEE	0.04	0.04	0.04
RMSECV	0.07	0.07	0.07
Permutation test			
R^2^ (min-max)	0.17–0.32	0.17–0.32	—
Q^2^ (min-max)	−2.28–−0.82	−2.28–−0.82	—
R^2^-intercept	0.22	0.22	0.22
Q^2^-intercept	−1.49	−1.49	−1.49

For the dendrogram of HCA displayed in Fig. 5B, it reveals that the inner part classes could be readily distinguished from the epidermis ones, which also verify the results of PLS-DA that different parts are also one of the factors that affect the chemical profiles of *W. cocos*. Additionally, for each part, the wild species could group into the same fraction firstly and then conjunctive to the cultivated ones, demonstrating the importance of growth patterns for the discrimination of samples. Briefly, it could be also inferred that the differences of chemical information in the two medicinal parts of *W. cocos* were much more than that in the same part with different growth patterns and geographical origins. In addition, combined with the results of PLS-DA score plot, it was possible to highlight that the growth patterns rather than the collection regions were the dominant factor for influencing the chemical properties of the same part samples, especially for the epidermis of *W. cocos*.

Up to now, in order to evaluate the properties of the raw materials of edible and medicinal mushrooms, many qualitative and quantitative techniques were established with the aid of chemometrics^15,17,36,37^. For the present study, the potential use of multi-spectral information fusion with the aid of chemometric methods as a tool to rapidly and comprehensively assess different properties of wild and cultivated *W. cocos* raw materials from diverse geographical origins has been successfully investigated. Totally, growth patterns, collection regions and different medicinal parts could affect the chemical properties of the samples and the last one was the most influential factor. Based on the previous studies, a number of chemical components of the inner part and epidermis of *W. cocos* were different including both the types and contents of the chemical compounds^19,32^. In addition, as recorded in Chinese Pharmacopoeia (versions 2015), the inner part and epidermis of *W. cocos* which had distinctively different clinical efficacies should be distinct usage in practice^18^. Our study confirmed the rationality of this principle from an overall chemical level.

On the other hand, with respect to edible and medicinal mushrooms, the lack of wild resources supply will eventually become a major bottleneck in the attempts for commercialization purposes, hence it is of paramount importance to establish a corresponding suitable cultivation methodology and the cultivated ones seem to be exploited as substitutes for the wild species^38,39^. However, not all of the cultivated materials could represent for the wild ones because growth patterns as well as external environment also play important roles in the variations of the chemical composition. As reported by Sari *et al*.^40^, wild-grown and commercially cultivated mushrooms were analyzed for their beta-glucan contents and the differences of samples were also measured. Moreover, Zhu *et al*.^41^ compared wild-grown high-quality *Ganoderma lucidum* with cultivated ones, and found the difference in the growth patterns among samples. According to Reis *et al*.^42^, it was reported that some wild-grown and cultivated edible mushrooms of the same species had different chemical composition, including fatty acids, sugars and tocopherols profiles. These researches above-mentioned were similar to the results of our study which suggested the wild and cultivated *W. cocos* could present differences between each other. In other words, the cultivated *W. cocos* was difficult to completely replace the wild species. During the growth periods, mushrooms could be influenced by the biotic and abiotic factors^43–45^. Nevertheless, compared with wild species, cultivation is aimed at providing the optimal growth conditions for mushrooms and normally the artificial management is necessary in this process. So, it is probably impacted by human activities for the cultivated mushrooms to some extent, which may be one reason for the differentiation between the wild and cultivated *W. cocos*. Prospectively, bionics wild cultivation may be a better way to narrow the differences between these two species.

What’s more, climate influences the growth and reproduction of species and changes in geographical locations are likely to affect the chemical properties of edible and medicinal mushrooms^46–50^. In terms of our previous researches, geographical origins seemed to be very important for *W. cocos*, not only the wild species but also the cultivated ones^51–54^. For this paper, even the raw material samples were originated from different growth patterns, geographic areas could also affect the chemical properties of *W. cocos*, which was similar to the former studies. Noteworthily, an interesting finding implied that growth patterns rather than the collection regions were likely to be the dominant factor for affecting the chemical components of the same part samples, especially for the epidermis of *W. cocos*, which also indicated better quality consistency of the inner parts. It may be due to the poorer resistance for external factors of epidermis than that of the inner parts. Furthermore, the inner parts are often covered by the epidermis, so the external part have more opportunity to be directly influenced by the external environment, which may cause the relative poor quality consistency of epidermis. However, more researches are needed to investigate the reasons.

## Conclusion

This work was a novel, yet interesting finding, as there have been no such studies on the differentiation of *W. cocos* raw materials using multi-spectral information fusion combined with chemometric methods. In conclusion, the variation of samples was generally correlated with their growth patterns, geographical origins as well as medicinal parts. However, medicinal parts played the most important role among the three factors that influenced the chemical properties of *W. cocos*. In addition, growth patterns rather than the collection regions were regarded as the dominant factor to effect the chemical files with respect to the same part samples, in particular for the epidermis ones. Overall, our findings were by no means exhaustive, but these results could provide qualitative interpretation and scientific supports to the claims on the differentiation of *W. cocos*. What’s more, it may provide a more comprehensive complement for the development and utilization of this species of edible and medicinal mushroom.

## Methods

### *W. cocos* samples and reagents

In total, 175 wild and cultivated fresh *W. cocos* sclerotia samples were collected from eight distinct areas (35 collection sites) in Yunnan Province of China (Table 5) and identified by Prof. Honggao Liu, Yunnan Agricultural University. Voucher specimens of all samples collected were deposited at Institute of Medicinal Plants, Yunnan Academy of Agricultural Sciences. The voucher specimens’ deposition numbers are presented in Table S6. After collection, the fresh samples were cleaned by a soft brush to remove any soil debris and air dried in the shade for about two months as well as further dried in an oven at 50 °C to a constant weight. Then, the inner part and epidermis of each sclerotium were separated, ground into powder and passed through a 100-mesh stainless steel sieve. The sieved powders were kept in new labeled Ziploc bags at room temperature prior to use. For the reagents, methanol of analytical grade from Xilong Chemical Company, Ltd. (China) and the potassium bromide (KBr) of spectroscopic grade were used.

**Table 5:** Information of *W. cocos* samples.

No	Code	Collection site	Part	Description	No	Code	Collection site	Part	Description
1–5	YX	Qinglongchang, Yuxi	Inner part	Wild	176–180	YX	Qinglongchang, Yuxi	Epidermis	Wild
6–10	YX	Yinyuan, Yuxi	Inner part	Wild	181–185	YX	Yinyuan, Yuxi	Epidermis	Wild
11–15	YX	Yangwu, Yuxi	Inner part	Wild	186–190	YX	Yangwu, Yuxi	Epidermis	Wild
16–20	YX	Dalongta, Yuxi	Inner part	Wild	191–195	YX	Dalongta, Yuxi	Epidermis	Wild
21–25	YX	Tadian, Yuxi	Inner part	Wild	196–200	YX	Tadian, Yuxi	Epidermis	Wild
26–30	YX	Dianzhong, Yuxi	Inner part	Wild	201–205	YX	Dianzhong, Yuxi	Epidermis	Wild
31–35	YX	Yaojie, Yuxi	Inner part	Wild	206–210	YX	Yaojie, Yuxi	Epidermis	Wild
36–40	YX	Xinping, Yuxi	Inner part	Wild	211–215	YX	Xinping, Yuxi	Epidermis	Wild
41–45	YX	Pingzhang, Yuxi	Inner part	Wild	216–220	YX	Pingzhang, Yuxi	Epidermis	Wild
46–50	WS	Matang, Wenshan	Inner part	Wild	221–225	WS	Matang, Wenshan	Epidermis	Wild
51–55	WS	Qiubei, Wenshan	Inner part	Wild	226–230	WS	Qiubei, Wenshan	Epidermis	Wild
56–60	WS	Bozhu, Wenshan	Inner part	Wild	231–235	WS	Bozhu, Wenshan	Epidermis	Wild
61–65	HH	Luxi, Honghe	Inner part	Wild	236–240	HH	Luxi, Honghe	Epidermis	Wild
66–70	HH	Mile, Honghe	Inner part	Wild	241–245	HH	Mile, Honghe	Epidermis	Wild
71–75	HH	Baoxiu, Honghe	Inner part	Wild	246–250	HH	Baoxiu, Honghe	Epidermis	Wild
76–80	HH	Kaiyuan, Honghe	Inner part	Wild	251–255	HH	Kaiyuan, Honghe	Epidermis	Wild
81–85	PE	Zhenyuan, Pu’er	Inner part	Wild	256–260	PE	Zhenyuan, Pu’er	Epidermis	Wild
86–90	PE	Linjie, Pu’er	Inner part	Wild	261–265	PE	Linjie, Pu’er	Epidermis	Wild
91–95	PE	Wenjing, Pu’er	Inner part	Wild	266–270	PE	Wenjing, Pu’er	Epidermis	Wild
96–100	CX	Bajiao, Chuxiong	Inner part	Wild	271–275	CX	Bajiao, Chuxiong	Epidermis	Wild
101–105	CX	Yijie, Chuxiong	Inner part	Wild	276–280	CX	Yijie, Chuxiong	Epidermis	Wild
106–110	CX	Xincun, Chuxiong	Inner part	Wild	281–285	CX	Xincun, Chuxiong	Epidermis	Wild
111–115	CX	Shuangbai, Chuxiong	Inner part	Wild	286–290	CX	Shuangbai, Chuxiong	Epidermis	Wild
116–120	CX	Hongtupo, Chuxiong	Inner part	Wild	291–295	CX	Hongtupo, Chuxiong	Epidermis	Wild
121–125	NJ	Lanping, Nujiang	Inner part	Wild	296–300	NJ	Lanping, Nujiang	Epidermis	Wild
126–130	NJ	Lanping, Nujiang	Inner part	Wild	301–305	NJ	Lanping, Nujiang	Epidermis	Wild
131–135	BS	Changning, Baoshan	Inner part	Cultivated	306–310	BS	Changning, Baoshan	Epidermis	Cultivated
136–140	BS	Tengchong, Baoshan	Inner part	Cultivated	311–315	BS	Tengchong, Baoshan	Epidermis	Cultivated
141–145	LC	Dawen, Lincang	Inner part	Cultivated	316–320	LC	Dawen, Lincang	Epidermis	Cultivated
146–150	LC	Mengmeng, Lincang	Inner part	Cultivated	321–325	LC	Mengmeng, Lincang	Epidermis	Cultivated
151–155	LC	Yongde, Lincang	Inner part	Cultivated	326–330	LC	Yongde, Lincang	Epidermis	Cultivated
156–160	PE	Zhenyuan, Pu’er	Inner part	Cultivated	331–335	PE	Zhenyuan, Pu’er	Epidermis	Cultivated
161–165	PE	Jinggu, Pu’er	Inner part	Cultivated	336–340	PE	Jinggu, Pu’er	Epidermis	Cultivated
166–170	PE	Mojiang, Pu’er	Inner part	Cultivated	341–345	PE	Mojiang, Pu’er	Epidermis	Cultivated
171–175	PE	Simao, Pu’er	Inner part	Cultivated	346–350	PE	Simao, Pu’er	Epidermis	Cultivated

### UV spectra acquisition

Each tested sample powder (0.5 g) was macerated for 30 min in 5.0 mL of methanol and extracted by ultrasonic extraction apparatus at 150 W for 60 min in a bath at 30 °C. The extracts were left to cool to room temperature and filtered through 0.22 μm membrane filters (Millipore, USA). Then 0.5 mL of the filtered solution was transferred to 25 mL volumetric flask and diluted with methanol for UV analysis. The spectra of *W. cocos* samples were immediately acquired using a TU-1901 PC UV-Visible Spectrophotometer (Pgeneral, Beijing, China) equipped with two 1 cm-thick quartz cells that one was for the extracts of samples and the other one was methanol in the wavelength range of 200–400 nm with a resolution of 2.0 nm, and the blank spectrum was recorded with methanol. Prior to each scan, baseline correction was also conducted. Each UV spectrum was measured in triplicate and all subsequent data analyses were performed by using the average spectrum of each triplicate. What’s more, the raw spectra were pretreated by standard normal variate (SNV) so as to correct light scatter and reduce the changes of light path length^55^.

### Mid-infrared spectroscopic analysis

1.5 mg of each *W. cocos* sample powder was mixed uniformly with spectroscopic grade KBr powder of 100 mg and then pressed into a pellet before mid-infrared spectral analysis. FTIR spectra were collected using a Frontier FTIR spectrophotometer (Perkin Elmer, USA) equipped with a deuterated triglycine sulfate (DTGS) detector at resolution of 4 cm^−1^ with 16 scans in the region of 4000–400 cm^−1^. Each spectrum with high signal-to-noise signal was recorded by an average of these 16 scans. All the measurements were carried out at room temperature. The sample spectra were obtained by subtraction of the background spectra measured using pure dried KBr in tablet form with the same parameters in order to remove the unwanted absorbance bands of carbon dioxide and water in the atmosphere. Each sample was recorded with three replicates and the average spectrum was used for further data analyses. Furthermore, all the original FTIR spectra were automatic baseline corrected and automatic smoothed using the Spectrum for Windows software (Nicolet OMNIC, Thermo Fisher Scientific) and also preprocessed by SNV.

### Spectral information fusion and statistical analysis

Competitive adaptive reweighted sampling (CARS) method is a simple but effective variable selection strategy and often used for selecting optimal combination of key wavelengths of multi-component spectral data based on four successive steps, including Monte Carlo model sampling, enforced wavelength reduction by exponentially decreasing function (EDF), competitive wavelength reduction by ARS and RMSECV calculation for each subset^56^. In this study, CARS was used to respectively select the key variables of preprocessed UV and FTIR spectra by using Matlab R2014a software (MathWorks, USA) with CARS toolkit (version 2.0). For each type of spectra, a total of 20 times samplings were respectively conducted and the variables which were selected more than two times were used to form a new data matrix that represented relevant characteristic spectral information. Then, based on the data fusion technique^10^, the new UV and FTIR data matrices were concatenated into a single matrix (fusion matrix) for next analyses. It also meant that the characteristic spectral information of these two types of spectra were integrated.

After spectral information fusion, the fusion matrices were subjected to establish PLS-DA models so as to carry out a detailed investigation among *W. cocos* samples on the basis of the growth patterns, geographical origins and parts. Generally, this method is on the basis of partial least squares regression, which transforms the observed data into a set of several intermediate linear latent variables (LVs) that are useful to predict the dependent variables which are so called class variables that indicate whether a given sample belongs to a given class^57^. So, it is necessary to have initial knowledge of the classes of tested samples. Moreover, in the application of PLS-DA procedure, binary encoding of designation for the class is used, where 1 means the object (sample) belongs to class and 0 doesn’t belong to class^58^. This assignment was used to establish the PLS-DA models and to test its prediction ability. For the construction of PLS-DA model, the fusion dataset was divided into two subsets, one used for calibration (two thirds of the samples) and the other used for external validation (consisting of the remaining samples) by Kennard-stone algorithm. Centering with no scaling (Ctr), which is the most commonly used scaling method for spectral data, was performed for the fusion matrices. The validation of the model was conducted by using 7-fold cross-validation and permutation tests (Y scrambling, n = 200). The performance of the model was evaluated in terms of some statistical parameters including R^2^Y, Q^2^, R^2^-intercept, Q^2^-intercept, sensitivity, specificity, efficiency, Matthews correlation coefficient (MCC), accuracy, root mean square error of estimation (RMSEE) and RMSECV. In particular, R^2^Y presents the cumulative interpretation ability and Q^2^ shows the cumulative prediction ability of the present model according to cross validation^59^. When the Q^2^ was >0.5, it suggested good predictive capacity of this method^60^. In addition, for the permutation test, if the R^2^ and Q^2^ values were less than 0.5 with scrambled dataset, and the intercept of R^2^ was below 0.4 as well as that of Q^2^ was below −0.05, the built PLS-DA model was considered as an appropriate model without overfitting because after mixing of activities data for conducting the permutation tests, the model lost its predictive power on the basis of the relationship between the variables was broken^61–63^. Moreover, sensitivity is defined as the fraction of the samples of modeled class accepted by the model while specificity describes the fraction of the samples in other class rejected by the model, and the efficiency presents the geometric mean of the sensitivity and specificity in order to summary the first two parameters of model performance^64^. Theoretically, index values of efficiency vary between 0 and 100%. The MCC is similar to the Person correlation coefficient that 1 suggests perfect classification, 0 indicates random classification as well as −1 means the worst possible prediction^65^. For the lowest RMSECV, it could guarantee the LVs collected as much as possible and they are not overfitted^66^. What’s more, HCA was applied to evaluate the degree of similarity among different classes of samples using the average fusion spectral information of each class. This technique is intended to create groups that maximize the cohesion internally and maximize separation externally^67^. Both PLS-DA and HCA were performed by SIMCA-P^+^ 13.0 (Umetrics, Umeå, Sweden).

## Electronic supplementary material

Supplementary Information
