# Supplementary material for: Differentiation and comparison of Wolfiporia cocos raw materials based on multi-spectral information fusion and chemometric methods
Source: Sci Rep. 2018 Aug 29;8:13043. doi: 10.1038/s41598-018-31264-1 (PMC6115471; doi:10.1038/s41598-018-31264-1)
Supplement: Supplementary file 1 — Supplementary Information [file 41598_2018_31264_MOESM1_ESM.doc]

**Differentiation and comparison of** ***Wolfiporia cocos* raw materials based on** **multi-spectral information fusion and** **chemometric methods**

**Yan Li & Yuanzhong Wang**

Institute of Medicinal Plants, Yunnan Academy of Agricultural Sciences, Kunming 650200, Yunnan, China

Correspondence should be addressed to Y.Z. Wang (e-mail: boletus@126.com)


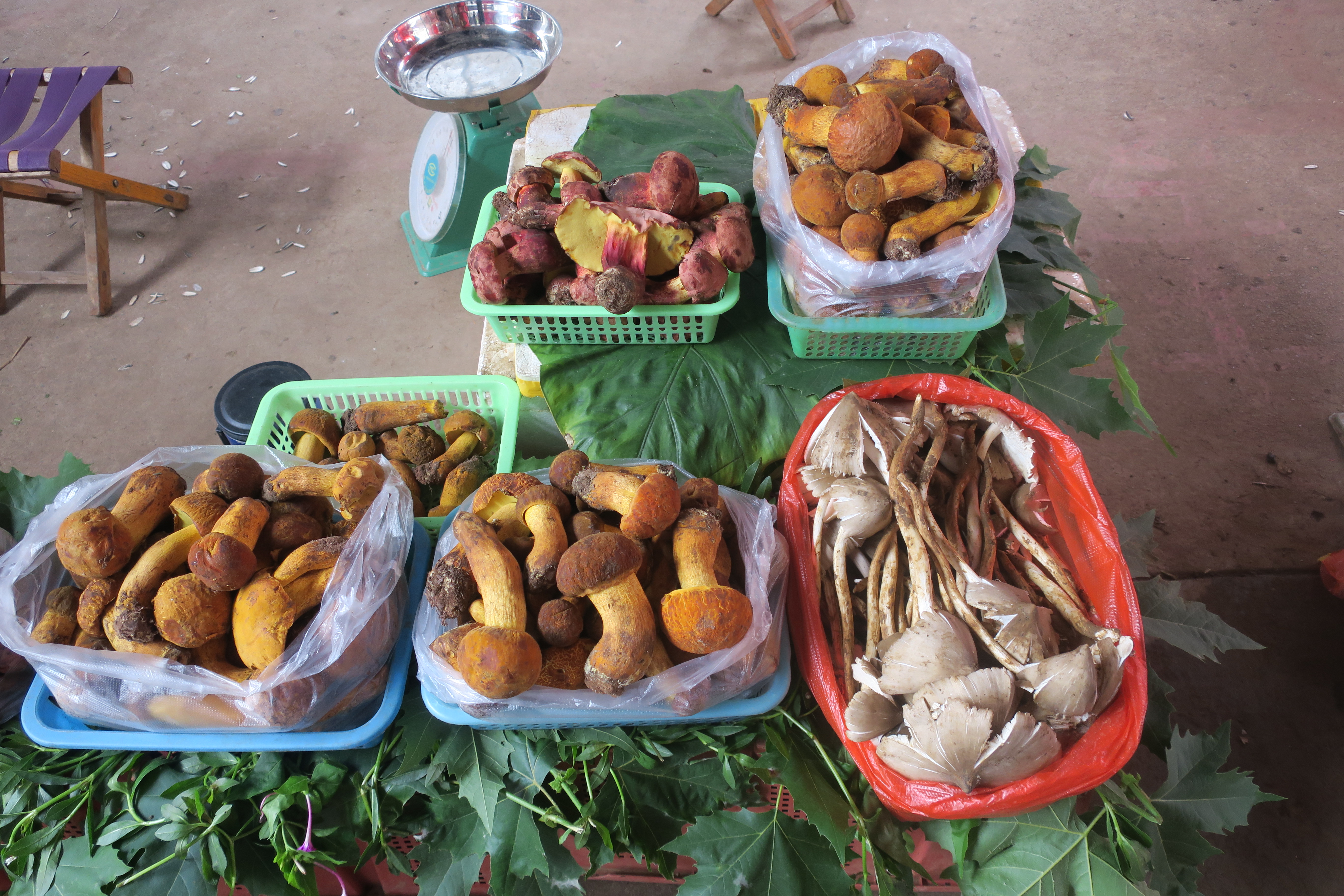


**Supplementary Figure S1.** Wild-grown mushrooms sold in the rural market.

**
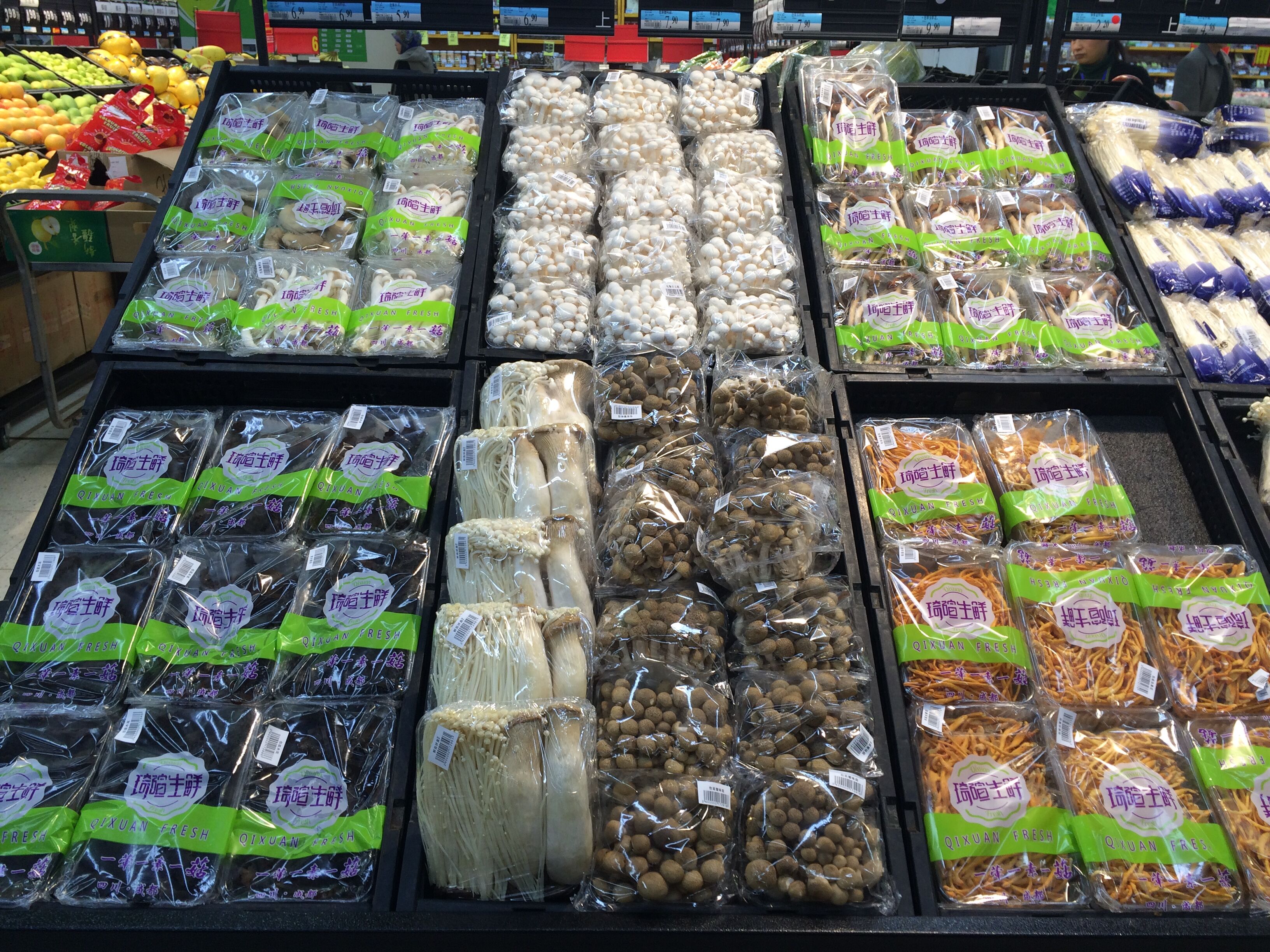
**

**Supplementary Figure S2.** Cultivated mushrooms sold in the market.

**
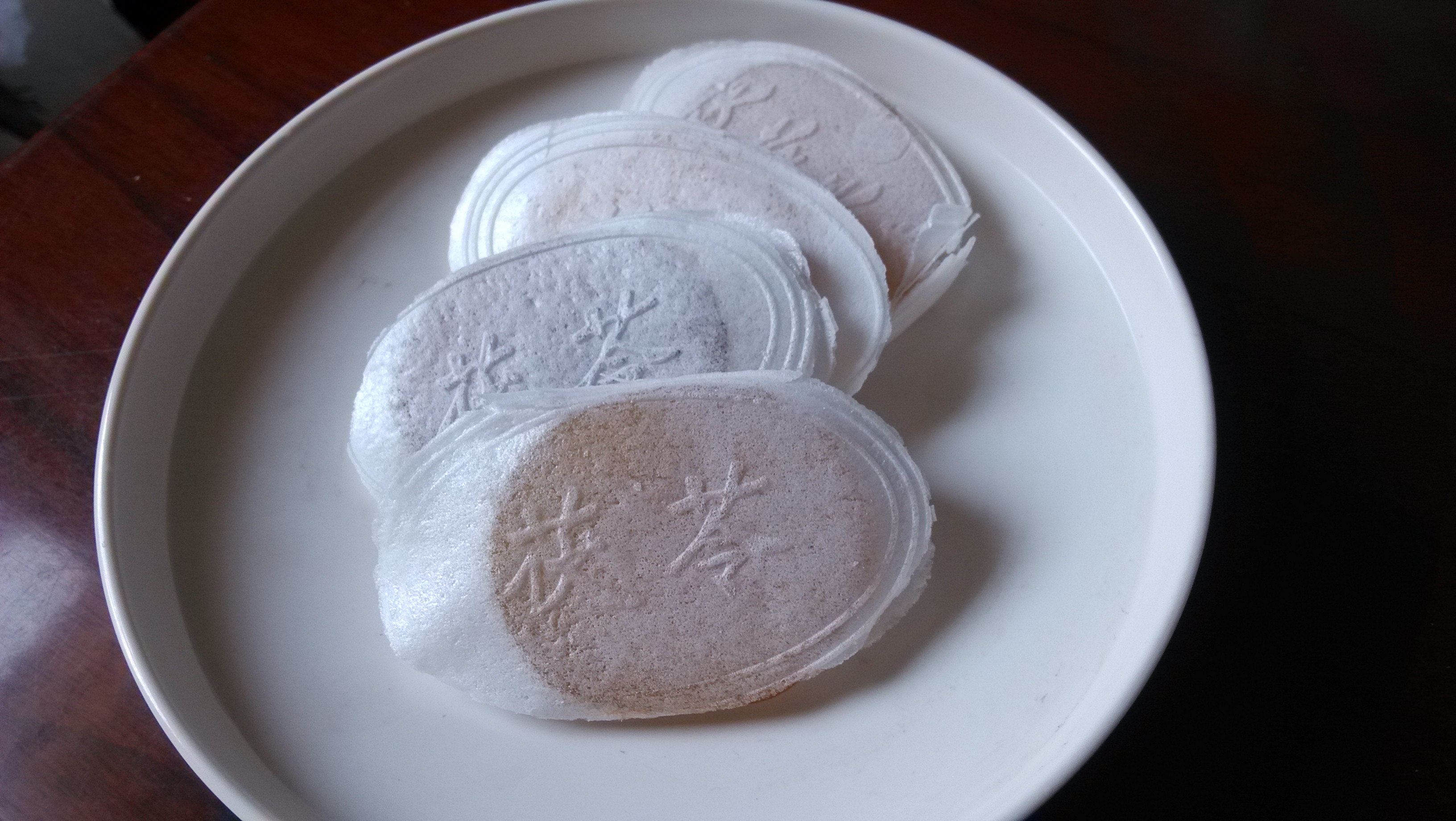
**

**Supplementary Figure S3.** Tuckahoe Pie, a traditional snack.

**
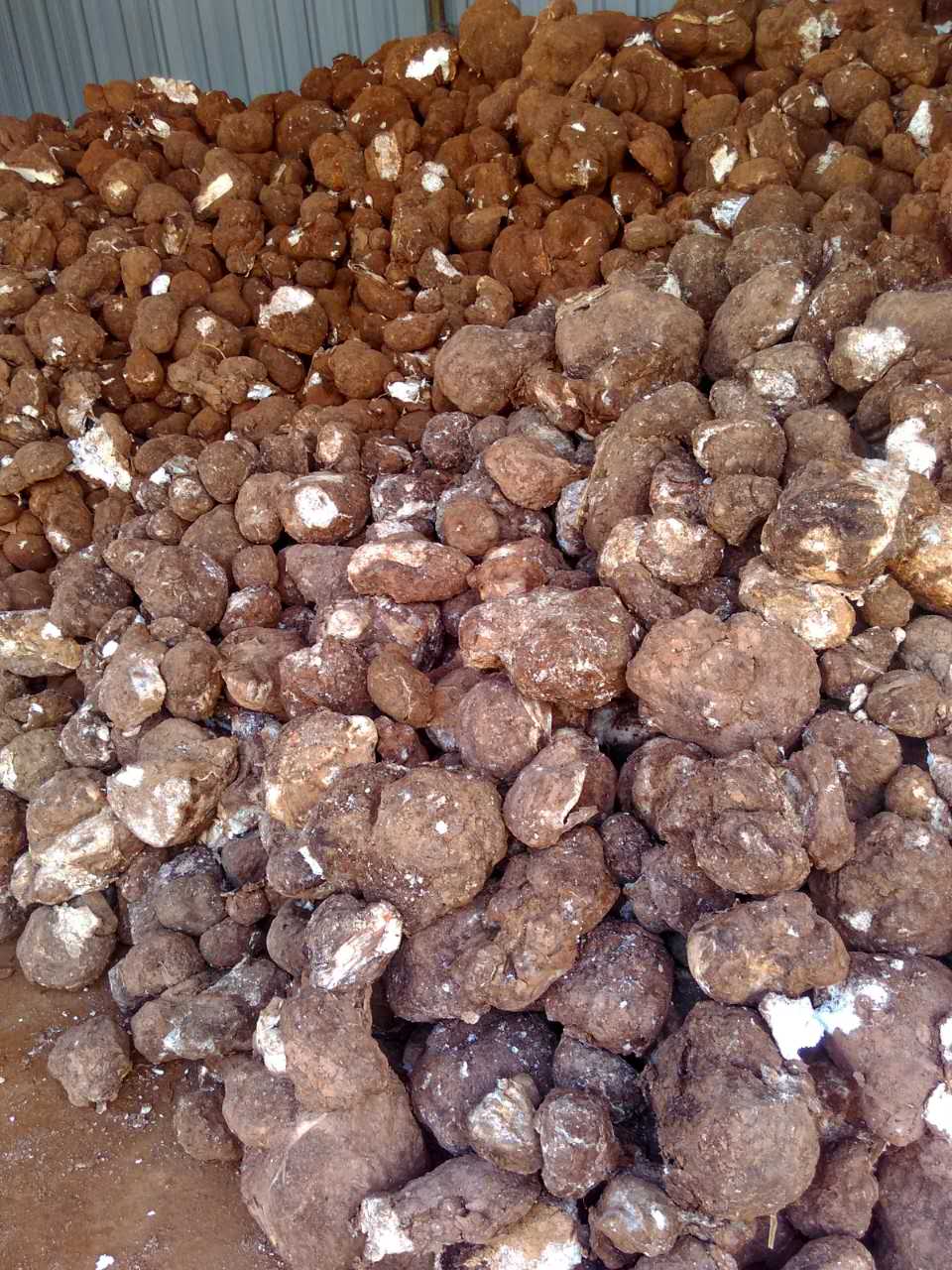
**

**Supplementary Figure S4.** Cultivated *Wolfiporia cocos*.

**Supplementary Table S1** VIPs of PLS-DA model based on the inner part samples with different growth patterns.

| UV | | | | |  | FTIR | | | | | | | |
| --- | --- | --- | --- | --- | --- | --- | --- | --- | --- | --- | --- | --- | --- |
| Wavelength (nm) | VIP |  | Wavelength (nm) | VIP |  | Wavenumber (cm^-1^) | VIP |  | Wavenumber (cm^-1^) | VIP |  | Wavenumber (cm^-1^) | VIP |
| **204.5** | 4.40427 |  | 302.5 | 0.36638 |  | 3694.94 | 0.93712 |  | 2030.68 | 0.39821 |  | 1284.36 | 0.78253 |
| **205** | 3.67044 |  | 303.5 | 0.32683 |  | 3693.01 | 0.95238 |  | 2028.75 | 0.40379 |  | 1282.43 | 0.79110 |
| **212.5** | 2.88199 |  | 306.5 | 0.46272 |  | **3685.3** | 1.01079 |  | 2026.82 | 0.40767 |  | 1280.5 | 0.80013 |
| **216.5** | 2.84354 |  | 309 | 0.37187 |  | **3554.16** | 1.12519 |  | 2024.89 | 0.41035 |  | 1259.29 | 0.80940 |
| **217.5** | 2.62525 |  | 314 | 0.31601 |  | **3546.45** | 1.13612 |  | 2022.96 | 0.41147 |  | 1257.36 | 0.80856 |
| **218** | 2.48484 |  | 316 | 0.28436 |  | **3542.59** | 1.14172 |  | 2021.03 | 0.41105 |  | 1255.43 | 0.80899 |
| **222.5** | 1.86388 |  | 316.5 | 0.28737 |  | **3540.67** | 1.14303 |  | 2019.1 | 0.40930 |  | 1253.5 | 0.81067 |
| **223** | 1.79579 |  | 318.5 | 0.39384 |  | **3536.81** | 1.14438 |  | 2017.18 | 0.40630 |  | 1251.57 | 0.81371 |
| **226.5** | 1.50907 |  | 325.5 | 0.35668 |  | **3457.74** | 1.27820 |  | 2009.46 | 0.40137 |  | 1249.65 | 0.81784 |
| **228.5** | 1.44612 |  | 327 | 0.38656 |  | **3455.81** | 1.27685 |  | 2007.53 | 0.40311 |  | 1247.72 | 0.82263 |
| **231** | 1.64193 |  | 327.5 | 0.39538 |  | **3448.1** | 1.27217 |  | 2005.61 | 0.40503 |  | 1245.79 | 0.82707 |
| **231.5** | 1.84542 |  | 328 | 0.40791 |  | 3340.1 | 0.85803 |  | 1997.89 | 0.40762 |  | 1243.86 | 0.83016 |
| **232.5** | 1.93048 |  | 328.5 | 0.50795 |  | 3336.25 | 0.83638 |  | 1995.96 | 0.40750 |  | 1234.22 | 0.86053 |
| **233.5** | 2.02398 |  | 329 | 0.34233 |  | 3334.32 | 0.82652 |  | 1994.03 | 0.40736 |  | 1232.29 | 0.87092 |
| **235** | 2.20136 |  | 330 | 0.27356 |  | 3330.46 | 0.80628 |  | 1749.12 | 0.24163 |  | 1230.36 | 0.88299 |
| **242** | 2.64656 |  | 332.5 | 0.30652 |  | 3328.53 | 0.79610 |  | 1747.19 | 0.25141 |  | 1228.43 | 0.89788 |
| **244.5** | 2.48544 |  | 333 | 0.19580 |  | 3326.61 | 0.78612 |  | 1745.26 | 0.26188 |  | 1180.22 | 0.96886 |
| **246** | 2.17256 |  | 334 | 0.25663 |  | 3324.68 | 0.77653 |  | 1743.33 | 0.27263 |  | 1178.29 | 0.95113 |
| **246.5** | 2.12085 |  | 337.5 | 0.49821 |  | 3322.75 | 0.76742 |  | 1741.41 | 0.28281 |  | 1176.36 | 0.93563 |
| **247** | 1.99977 |  | 338 | 0.38428 |  | 3320.82 | 0.75867 |  | 1739.48 | 0.29365 |  | 1174.44 | 0.92088 |
| **250** | 1.86932 |  | 340 | 0.33929 |  | 3318.89 | 0.75021 |  | 1708.62 | 0.55991 |  | 1172.51 | 0.90367 |
| **252.5** | 1.68764 |  | 345 | 0.36325 |  | 3316.96 | 0.74205 |  | 1706.69 | 0.58294 |  | 1170.58 | 0.88129 |
| **253** | 1.58353 |  | 346 | 0.31228 |  | 3315.03 | 0.73411 |  | 1704.76 | 0.60579 |  | 1168.65 | 0.85020 |
| 256 | 0.90867 |  | 349.5 | 0.31972 |  | 3313.11 | 0.72622 |  | 1702.84 | 0.62896 |  | 1166.72 | 0.80830 |
| 263.5 | 0.65697 |  | 350 | 0.29291 |  | 3311.18 | 0.71842 |  | 1700.91 | 0.65337 |  | 1130.08 | 0.62219 |
| 264 | 0.61959 |  | 351 | 0.40887 |  | 3309.25 | 0.71064 |  | 1698.98 | 0.67917 |  | 1128.15 | 0.63899 |
| 265 | 0.62670 |  | 352 | 0.46623 |  | 2915.84 | 0.90768 |  | 1697.05 | 0.70621 |  | 1108.87 | 0.74748 |
| 266.5 | 0.60890 |  | 352.5 | 0.42074 |  | 2913.91 | 0.90773 |  | 1695.12 | 0.73591 |  | 1106.94 | 0.74890 |
| 267 | 0.76557 |  | 353 | 0.43530 |  | 2911.98 | 0.90817 |  | **1666.2** | 1.39194 |  | 1105.01 | 0.74676 |
| 268 | 0.77894 |  | 354.5 | 0.58438 |  | 2910.06 | 0.90915 |  | **1664.27** | 1.40936 |  | 1103.08 | 0.74438 |
| 268.5 | 0.73389 |  | 355 | 0.44664 |  | 2908.13 | 0.91070 |  | **1662.34** | 1.41407 |  | **1074.16** | 1.15229 |
| 269 | 0.88004 |  | 355.5 | 0.52690 |  | 2906.2 | 0.91254 |  | **1660.41** | 1.40742 |  | **1072.23** | 1.12260 |
| 270 | 0.81500 |  | 359.5 | 0.44578 |  | 2865.7 | 0.88921 |  | **1658.48** | 1.39291 |  | **1070.3** | 1.08090 |
| 271 | 0.83889 |  | 360 | 0.50275 |  | 2863.77 | 0.88453 |  | **1656.55** | 1.36982 |  | **1068.37** | 1.03191 |
| 272 | 0.83174 |  | 361 | 0.33396 |  | 2861.84 | 0.87972 |  | **1654.62** | 1.34153 |  | 1066.44 | 0.98121 |
| 274 | 0.91444 |  | 361.5 | 0.47679 |  | 2857.99 | 0.86942 |  | **1621.84** | 1.49515 |  | **997.017** | 1.04208 |
| 275 | 0.90506 |  | 363 | 0.47555 |  | 2856.06 | 0.86460 |  | **1619.91** | 1.48399 |  | **995.089** | 1.06576 |
| **276** | 1.01687 |  | 363.5 | 0.32204 |  | 2854.13 | 0.86039 |  | **1617.98** | 1.45271 |  | **993.16** | 1.08575 |
| 276.5 | 0.93294 |  | 366 | 0.34390 |  | 2852.2 | 0.85651 |  | 1598.7 | 0.86556 |  | **991.232** | 1.09650 |
| **279** | 1.02926 |  | 367 | 0.32962 |  | 2850.27 | 0.85254 |  | 1596.77 | 0.85230 |  | **989.303** | 1.09293 |
| **279.5** | 1.24427 |  | 369.5 | 0.41109 |  | 2848.35 | 0.84825 |  | 1594.84 | 0.84913 |  | 898.666 | 0.78146 |
| **280.5** | 1.19329 |  | 370 | 0.44354 |  | 2846.42 | 0.84352 |  | 1587.13 | 0.93782 |  | 896.737 | 0.76751 |
| **283.5** | 1.15814 |  | 370.5 | 0.29865 |  | 2844.49 | 0.83839 |  | 1585.2 | 0.97850 |  | 894.809 | 0.76652 |
| **284.5** | 1.18874 |  | 374.5 | 0.36978 |  | 2840.63 | 0.82692 |  | **1565.92** | 1.35094 |  | 892.88 | 0.77929 |
| **285.5** | 1.11609 |  | 375 | 0.52019 |  | 2327.66 | 0.44821 |  | **1563.99** | 1.36084 |  | 889.023 | 0.83247 |
| **286.5** | 1.09595 |  | 382 | 0.54646 |  | 2325.73 | 0.44768 |  | **1562.06** | 1.36299 |  | 887.095 | 0.86166 |
| **287** | 1.03442 |  | 383 | 0.42071 |  | 2323.8 | 0.44778 |  | 1425.14 | 0.79426 |  | 769.458 | 0.79096 |
| **287.5** | 1.01258 |  | 385.5 | 0.52972 |  | 2321.87 | 0.44808 |  | 1423.21 | 0.78675 |  | 740.531 | 0.82050 |
| 288 | 0.93047 |  | 386 | 0.44554 |  | 2319.95 | 0.44801 |  | 1421.28 | 0.77919 |  | 738.603 | 0.82110 |
| 289.5 | 0.96688 |  | 388 | 0.46410 |  | 2318.02 | 0.44762 |  | 1413.57 | 0.74637 |  | 698.105 | 0.66607 |
| 290.5 | 0.93626 |  | 389.5 | 0.36263 |  | 2316.09 | 0.44716 |  | 1411.64 | 0.73820 |  | 474.403 | 0.76551 |
| **291** | 1.11993 |  | 391.5 | 0.48295 |  | 2314.16 | 0.44609 |  | 1373.07 | 0.76295 |  | 472.474 | 0.78662 |
| **292.5** | 1.05309 |  | 392.5 | 0.38638 |  | 2073.1 | 0.35784 |  | 1371.14 | 0.77902 |  | 404.978 | 0.59900 |
| **293** | 1.10065 |  | 393.5 | 0.41584 |  | 2061.53 | 0.35979 |  | **1330.64** | 1.10367 |  | 403.05 | 0.59689 |
| 293.5 | 0.99570 |  | 395.5 | 0.37833 |  | 2059.6 | 0.36033 |  | **1328.71** | 1.15542 |  |  |  |
| **294.5** | 1.03197 |  | 396 | 0.40183 |  | 2057.67 | 0.36110 |  | **1326.79** | 1.21576 |  |  |  |
| 295 | 0.99730 |  | 397 | 0.35941 |  | 2048.03 | 0.36642 |  | 1295.93 | 0.76883 |  |  |  |
| 295.5 | 0.86913 |  | 397.5 | 0.46208 |  | 2046.1 | 0.36742 |  | 1294 | 0.76525 |  |  |  |
| 297 | 0.65975 |  | 399.5 | 0.45321 |  | 2044.17 | 0.36869 |  | 1292.07 | 0.76406 |  |  |  |
| 300.5 | 0.46772 |  | 400 | 0.52504 |  | 2034.53 | 0.38348 |  | 1288.22 | 0.76925 |  |  |  |
| 302 | 0.34552 |  |  |  |  | 2032.6 | 0.39090 |  | 1286.29 | 0.77506 |  |  |  |

Notes: the variables with VIP scores greater than 1 are formatted in bold.

**Supplementary Table S2** VIPs of PLS-DA model based on the epidermis samples with different growth patterns.

| UV | | | | |  | FTIR | | | | |
| --- | --- | --- | --- | --- | --- | --- | --- | --- | --- | --- |
| Wavelength (nm) | VIP |  | Wavelength (nm) | VIP |  | Wavenumber (cm^-1^) | VIP |  | Wavenumber (cm^-1^) | VIP |
| **200.5** | 1.67498 |  | 310.5 | 0.07131 |  | **3710.37** | 1.09268 |  | 1567.84 | 0.469017 |
| **201** | 1.97242 |  | 311 | 0.08559 |  | **3708.44** | 1.1316 |  | 1565.92 | 0.443914 |
| **202** | 2.49868 |  | 314.5 | 0.07555 |  | **3706.51** | 1.1704 |  | 1563.99 | 0.420084 |
| **202.5** | 2.67484 |  | 315 | 0.07840 |  | **3704.58** | 1.20651 |  | 1562.06 | 0.397507 |
| **203.5** | 2.67453 |  | 315.5 | 0.06843 |  | **3702.66** | 1.23793 |  | 1560.13 | 0.374667 |
| 211 | 0.89404 |  | 316 | 0.08225 |  | **3700.73** | 1.26259 |  | 1542.77 | 0.312794 |
| 213.5 | 0.93276 |  | 318 | 0.07477 |  | **3698.8** | 1.2791 |  | 1540.84 | 0.33582 |
| 214.5 | 0.91765 |  | 318.5 | 0.09232 |  | **3696.87** | 1.28688 |  | 1538.92 | 0.359905 |
| 215 | 0.91520 |  | 319 | 0.06917 |  | **3694.94** | 1.28693 |  | 1536.99 | 0.382285 |
| 215.5 | 0.90303 |  | 321.5 | 0.06341 |  | **3693.01** | 1.28075 |  | 1535.06 | 0.403555 |
| 216 | 0.89187 |  | 322 | 0.07892 |  | **3691.09** | 1.27033 |  | 1533.13 | 0.422066 |
| 216.5 | 0.88002 |  | 322.5 | 0.07366 |  | **3689.16** | 1.25882 |  | 1531.2 | 0.434879 |
| 217 | 0.86181 |  | 323 | 0.07186 |  | **3687.23** | 1.24864 |  | 1529.27 | 0.442279 |
| 220 | 0.72036 |  | 323.5 | 0.08379 |  | **3685.3** | 1.24297 |  | 1527.35 | 0.445081 |
| 223 | 0.57782 |  | 324 | 0.06879 |  | **3648.66** | 1.38934 |  | 1525.42 | 0.442885 |
| 225.5 | 0.50008 |  | 325 | 0.07173 |  | **3646.73** | 1.38856 |  | 1523.49 | 0.437493 |
| 227 | 0.47236 |  | 326 | 0.07434 |  | **3644.8** | 1.38733 |  | 1521.56 | 0.4281 |
| 229 | 0.48636 |  | 326.5 | 0.06634 |  | **3642.87** | 1.38627 |  | 1382.71 | 0.519077 |
| 231.5 | 0.55279 |  | 327 | 0.07943 |  | **3637.09** | 1.38231 |  | 1380.78 | 0.533975 |
| 234 | 0.58750 |  | 327.5 | 0.07309 |  | **3615.88** | 1.3021 |  | 1378.85 | 0.550508 |
| 234.5 | 0.57287 |  | 328 | 0.07877 |  | **3613.95** | 1.27549 |  | 1376.93 | 0.568616 |
| 235 | 0.64116 |  | 328.5 | 0.07684 |  | **3612.02** | 1.24903 |  | 1375 | 0.587123 |
| 238.5 | 0.73539 |  | 329 | 0.07838 |  | **3610.09** | 1.22347 |  | 1369.21 | 0.6055 |
| 239.5 | 0.94635 |  | 329.5 | 0.08094 |  | **3608.16** | 1.19939 |  | 1367.28 | 0.596876 |
| 240 | 0.98184 |  | 330 | 0.07472 |  | **3604.3** | 1.15682 |  | 1365.35 | 0.587535 |
| **242** | 1.51919 |  | 332.5 | 0.09260 |  | **3471.24** | 1.84179 |  | 1363.43 | 0.577983 |
| 245 | 0.61800 |  | 336 | 0.10233 |  | **3469.31** | 1.86047 |  | 1361.5 | 0.568012 |
| 245.5 | 0.69407 |  | 337.5 | 0.08549 |  | **3467.38** | 1.8791 |  | 1359.57 | 0.557928 |
| 246 | 0.70222 |  | 338.5 | 0.08800 |  | **3465.45** | 1.89738 |  | 1357.64 | 0.548073 |
| 247.5 | 0.62139 |  | 340 | 0.08546 |  | **3463.53** | 1.91564 |  | 1355.71 | 0.537957 |
| 248 | 0.60061 |  | 341 | 0.09170 |  | **3357.46** | 1.58369 |  | 1261.22 | 0.684788 |
| 248.5 | 0.59330 |  | 342 | 0.08768 |  | **3355.53** | 1.56587 |  | 1259.29 | 0.691497 |
| 249 | 0.57174 |  | 342.5 | 0.09668 |  | **3353.6** | 1.54768 |  | 1257.36 | 0.697441 |
| 249.5 | 0.56743 |  | 343 | 0.09301 |  | **3351.68** | 1.52927 |  | 1255.43 | 0.702821 |
| 250.5 | 0.55216 |  | 344.5 | 0.09632 |  | **3349.75** | 1.51067 |  | 1253.5 | 0.707705 |
| 251 | 0.53588 |  | 350 | 0.11419 |  | **3347.82** | 1.49185 |  | 1251.57 | 0.711909 |
| 251.5 | 0.52408 |  | 352 | 0.12419 |  | **3345.89** | 1.47287 |  | 1249.65 | 0.715967 |
| 254.5 | 0.39195 |  | 353 | 0.12253 |  | **3343.96** | 1.45398 |  | **1143.58** | 1.40245 |
| 255.5 | 0.29612 |  | 355 | 0.12363 |  | **3342.03** | 1.43543 |  | **1141.65** | 1.4414 |
| 256.5 | 0.23745 |  | 356.5 | 0.12496 |  | **3340.1** | 1.41704 |  | **1139.72** | 1.48635 |
| 257.5 | 0.25085 |  | 358 | 0.12127 |  | **3338.18** | 1.39824 |  | **1137.8** | 1.53669 |
| 259 | 0.31503 |  | 359 | 0.12556 |  | **3336.25** | 1.3785 |  | **1135.87** | 1.59141 |
| 260 | 0.34773 |  | 360.5 | 0.12435 |  | **3334.32** | 1.35825 |  | **1133.94** | 1.64889 |
| 261 | 0.37243 |  | 367.5 | 0.13882 |  | **3332.39** | 1.33811 |  | **1132.01** | 1.70786 |
| 262.5 | 0.38530 |  | 368 | 0.13460 |  | **3330.46** | 1.31806 |  | **1130.08** | 1.7665 |
| 263 | 0.37331 |  | 369 | 0.13647 |  | **3326.61** | 1.27755 |  | **1128.15** | 1.82239 |
| 263.5 | 0.37343 |  | 369.5 | 0.14437 |  | **3322.75** | 1.23682 |  | **1126.22** | 1.87366 |
| 264 | 0.36073 |  | 370.5 | 0.14256 |  | **3160.76** | 1.05814 |  | **1124.3** | 1.91906 |
| 266 | 0.33445 |  | 371.5 | 0.14256 |  | **3158.83** | 1.0644 |  | **1049.09** | 1.72594 |
| 266.5 | 0.31975 |  | 372.5 | 0.14406 |  | **3156.9** | 1.07013 |  | **1047.16** | 1.72387 |
| 267.5 | 0.31319 |  | 373.5 | 0.14888 |  | **3154.97** | 1.07517 |  | **1045.23** | 1.72098 |
| 268.5 | 0.30053 |  | 374.5 | 0.16132 |  | **3153.04** | 1.07964 |  | **1018.23** | 1.71292 |
| 269.5 | 0.29002 |  | 375 | 0.14898 |  | **3151.11** | 1.08394 |  | **1016.3** | 1.73991 |
| 275 | 0.27797 |  | 377.5 | 0.15599 |  | **3149.19** | 1.08808 |  | **1014.37** | 1.77321 |
| 276 | 0.27958 |  | 378.5 | 0.15749 |  | **3147.26** | 1.09168 |  | **1012.45** | 1.81016 |
| 277.5 | 0.28876 |  | 382.5 | 0.18717 |  | **3145.33** | 1.09448 |  | **975.804** | 1.95331 |
| 283 | 0.32756 |  | 383.5 | 0.19356 |  | **3143.4** | 1.09663 |  | **971.947** | 1.81401 |
| 284 | 0.33300 |  | 385.5 | 0.18950 |  | **3141.47** | 1.09848 |  | **970.019** | 1.73672 |
| 285 | 0.32041 |  | 386.5 | 0.19450 |  | **3137.62** | 1.10173 |  | **948.806** | 1.00915 |
| 285.5 | 0.32522 |  | 387 | 0.21638 |  | 1762.62 | 0.179701 |  | 946.877 | 0.975707 |
| 286 | 0.31852 |  | 388.5 | 0.21249 |  | 1760.69 | 0.190342 |  | 944.949 | 0.949086 |
| 286.5 | 0.31580 |  | 389.5 | 0.23395 |  | 1758.76 | 0.202704 |  | 943.02 | 0.92837 |
| 287.5 | 0.31041 |  | 390.5 | 0.24534 |  | 1756.83 | 0.216045 |  | 941.092 | 0.912793 |
| 289 | 0.29733 |  | 392 | 0.24097 |  | 1754.9 | 0.230282 |  | **829.241** | 1.52442 |
| 290 | 0.29459 |  | 393 | 0.24931 |  | 1716.34 | 0.674072 |  | **827.312** | 1.52276 |
| 291 | 0.28737 |  | 393.5 | 0.24428 |  | 1714.41 | 0.698309 |  | **825.384** | 1.51534 |
| 292.5 | 0.27208 |  | 394 | 0.25593 |  | 1712.48 | 0.721386 |  | 769.458 | 0.968851 |
| 296.5 | 0.20351 |  | 395 | 0.25671 |  | 1710.55 | 0.739162 |  | 767.53 | 0.966122 |
| 297.5 | 0.19067 |  | 395.5 | 0.26997 |  | 1708.62 | 0.752795 |  | 765.601 | 0.960677 |
| 298 | 0.18417 |  | 396.5 | 0.27190 |  | 1706.69 | 0.760202 |  | 763.673 | 0.955942 |
| 298.5 | 0.17598 |  | 397.5 | 0.27398 |  | 1683.55 | 0.878909 |  | 761.744 | 0.954506 |
| 299.5 | 0.16048 |  | 398.5 | 0.28391 |  | 1681.62 | 0.93754 |  | 759.816 | 0.956576 |
| 300 | 0.14935 |  | 400 | 0.28184 |  | **1679.69** | 1.0078 |  | **474.403** | 2.14907 |
| 300.5 | 0.14094 |  |  |  |  | **1677.77** | 1.08825 |  | **472.474** | 2.20614 |
| 301 | 0.13471 |  |  |  |  | **1675.84** | 1.17596 |  | **470.546** | 2.24146 |
| 302 | 0.12903 |  |  |  |  | **1673.91** | 1.26945 |  | **453.19** | 1.80735 |
| 302.5 | 0.11323 |  |  |  |  | **1671.98** | 1.36416 |  | **451.261** | 1.73243 |
| 303 | 0.11326 |  |  |  |  | **1619.91** | 2.80489 |  | **449.333** | 1.6623 |
| 303.5 | 0.10850 |  |  |  |  | **1617.98** | 2.73657 |  | **447.404** | 1.60184 |
| 304 | 0.10529 |  |  |  |  | **1616.06** | 2.62632 |  | **445.476** | 1.55378 |
| 305 | 0.09431 |  |  |  |  | **1614.13** | 2.48018 |  | **443.547** | 1.52059 |
| 306 | 0.08814 |  |  |  |  | 1577.49 | 0.601751 |  | **416.549** | 1.56813 |
| 306.5 | 0.08456 |  |  |  |  | 1575.56 | 0.571123 |  | **414.62** | 1.60914 |
| 308 | 0.07964 |  |  |  |  | 1573.63 | 0.542281 |  | **412.692** | 1.65224 |
| 309 | 0.07995 |  |  |  |  | 1571.7 | 0.518755 |  | **410.763** | 1.6992 |
| 310 | 0.08034 |  |  |  |  | 1569.77 | 0.494787 |  |  |  |

Notes: the variables with VIP scores greater than 1 are formatted in bold.

**Supplementary Table S3** VIPs of PLS-DA modeling on the inner parts of *W. cocos* with different collection regions.

| UV | |  | FTIR | | | | |
| --- | --- | --- | --- | --- | --- | --- | --- |
| Wavelength (nm) | VIP |  | Wavenumber (cm^-1^) | VIP |  | Wavenumber (cm^-1^) | VIP |
| **203** | 3.47732 |  | 3905.15 | 0.47450 |  | 1535.06 | 0.62339 |
| **203.5** | 3.03831 |  | 3901.29 | 0.50119 |  | 1533.13 | 0.61845 |
| **205.5** | 2.11470 |  | 3899.36 | 0.51424 |  | 1531.2 | 0.61319 |
| **207.5** | 2.18884 |  | 3862.72 | 0.57615 |  | 1529.27 | 0.60800 |
| **208.5** | 2.07798 |  | 3860.79 | 0.59188 |  | 1513.85 | 0.66106 |
| **210** | 1.94368 |  | 3858.86 | 0.60038 |  | 1511.92 | 0.67229 |
| **213.5** | 1.59853 |  | 3856.93 | 0.60353 |  | 1509.99 | 0.68326 |
| **215** | 1.50845 |  | 3855.01 | 0.60054 |  | 1508.06 | 0.69240 |
| **215.5** | 1.41359 |  | 3853.08 | 0.59947 |  | 1506.13 | 0.69575 |
| **217** | 1.27597 |  | 3785.58 | 0.67651 |  | 1504.2 | 0.69840 |
| **218.5** | 1.17704 |  | 3783.65 | 0.68812 |  | 1490.7 | 0.74851 |
| **220.5** | 1.51734 |  | 3781.72 | 0.69849 |  | 1488.78 | 0.74818 |
| **221** | 1.29395 |  | 3268.75 | 0.58253 |  | 1486.85 | 0.74642 |
| **222** | 1.32534 |  | 3266.82 | 0.58288 |  | 1484.92 | 0.74392 |
| **222.5** | 1.37881 |  | 3264.89 | 0.58396 |  | 1482.99 | 0.74434 |
| **226** | 1.25352 |  | 3262.97 | 0.58556 |  | **1382.71** | 1.11327 |
| **226.5** | 1.17765 |  | 3261.04 | 0.58672 |  | **1380.78** | 1.06394 |
| **227.5** | 1.43822 |  | 3259.11 | 0.58633 |  | 1361.5 | 0.61413 |
| **228** | 1.26694 |  | 3257.18 | 0.58394 |  | 1359.57 | 0.60134 |
| **229** | 1.29339 |  | 3255.25 | 0.58078 |  | 1357.64 | 0.59685 |
| **232** | 1.15197 |  | 3253.32 | 0.57819 |  | 1355.71 | 0.59462 |
| **232.5** | 1.27235 |  | 3251.4 | 0.57694 |  | 1353.78 | 0.59162 |
| **234.5** | 1.16795 |  | 3249.47 | 0.57739 |  | 1351.86 | 0.58636 |
| **236** | 1.18905 |  | 3247.54 | 0.57884 |  | 1349.93 | 0.58022 |
| **240** | 1.46723 |  | 3245.61 | 0.57997 |  | 1348 | 0.57981 |
| **241** | 1.35706 |  | 3241.75 | 0.58061 |  | 1346.07 | 0.58965 |
| **242** | 1.48780 |  | 3235.97 | 0.58813 |  | 1334.5 | 0.69419 |
| **246.5** | 1.33329 |  | 3149.19 | 0.91779 |  | 1332.57 | 0.72398 |
| **248** | 1.16665 |  | 3126.04 | 0.97726 |  | 1330.64 | 0.76434 |
| **248.5** | 1.42057 |  | 3118.33 | 0.98734 |  | 1328.71 | 0.82025 |
| 249 | 0.96288 |  | 3116.4 | 0.99061 |  | 1326.79 | 0.89377 |
| 250 | 0.93201 |  | 3114.47 | 0.99459 |  | 1324.86 | 0.98134 |
| **253.5** | 1.14935 |  | 3112.55 | 0.99937 |  | 1263.15 | 0.62075 |
| **254.5** | 1.09083 |  | 2965.98 | 0.97222 |  | 1261.22 | 0.62141 |
| **255.5** | 1.08136 |  | 2915.84 | 0.73289 |  | 1259.29 | 0.62599 |
| **256** | 1.12263 |  | 2913.91 | 0.71229 |  | 1257.36 | 0.63604 |
| **259** | 1.41353 |  | 2911.98 | 0.69472 |  | 1255.43 | 0.65353 |
| **260.5** | 1.30163 |  | 2910.06 | 0.68050 |  | 1157.08 | 0.82498 |
| **261.5** | 1.20930 |  | 2908.13 | 0.66965 |  | 1155.15 | 0.77463 |
| **262** | 1.42334 |  | 2906.2 | 0.66169 |  | 1153.22 | 0.73794 |
| **268** | 1.41131 |  | 2904.27 | 0.65618 |  | 1151.29 | 0.71366 |
| **273.5** | 1.23691 |  | 2902.34 | 0.65263 |  | 1149.37 | 0.70275 |
| **275.5** | 1.11631 |  | 2896.56 | 0.65232 |  | 1147.44 | 0.70801 |
| **280** | 1.20865 |  | 2894.63 | 0.65402 |  | 1145.51 | 0.72479 |
| **282** | 1.52100 |  | 2892.7 | 0.65560 |  | 1143.58 | 0.74552 |
| **288** | 1.40378 |  | 2886.91 | 0.65946 |  | 1141.65 | 0.76576 |
| **294.5** | 1.24353 |  | 2884.99 | 0.66034 |  | **1076.08** | 1.82454 |
| 300 | 0.75488 |  | 2883.06 | 0.66035 |  | **1074.16** | 1.79511 |
| 302.5 | 0.82179 |  | 2881.13 | 0.65931 |  | **1072.23** | 1.74548 |
| 303.5 | 0.65035 |  | 2842.56 | 0.66090 |  | **1022.09** | 1.41383 |
| 304 | 0.92791 |  | 2838.7 | 0.65872 |  | **987.375** | 1.24674 |
| 304.5 | 0.63533 |  | 2836.77 | 0.65809 |  | **985.447** | 1.25400 |
| 305 | 0.96389 |  | 2834.85 | 0.65800 |  | **983.518** | 1.26551 |
| 305.5 | 0.73949 |  | 2832.92 | 0.65761 |  | **981.59** | 1.28564 |
| **310** | 1.01290 |  | 2830.99 | 0.65641 |  | **979.661** | 1.31635 |
| 310.5 | 0.96980 |  | 2719.14 | 0.52946 |  | **977.733** | 1.35035 |
| **311** | 1.11334 |  | 2707.57 | 0.51820 |  | **944.949** | 1.44933 |
| 317 | 0.74516 |  | 2634.29 | 0.45032 |  | **943.02** | 1.42808 |
| **317.5** | 1.22253 |  | 2620.79 | 0.43908 |  | **941.092** | 1.40305 |
| 319 | 0.99240 |  | 2368.16 | 0.42241 |  | **933.378** | 1.26927 |
| 320 | 0.99485 |  | 2366.23 | 0.43206 |  | **931.449** | 1.23454 |
| 323.5 | 0.84217 |  | 2364.3 | 0.43674 |  | **929.521** | 1.20262 |
| 332.5 | 0.92119 |  | 1774.19 | 0.49801 |  | 806.099 | 0.74512 |
| **339.5** | 1.10597 |  | 1772.26 | 0.50650 |  | 673.035 | 0.74363 |
| 340.5 | 0.91792 |  | 1770.33 | 0.51029 |  | 671.106 | 0.74052 |
| 343 | 0.98321 |  | 1768.4 | 0.51141 |  | 669.178 | 0.74367 |
| 343.5 | 0.96999 |  | 1752.98 | 0.55328 |  | 667.25 | 0.75430 |
| 345 | 0.76144 |  | 1751.05 | 0.56792 |  | 665.321 | 0.76545 |
| 345.5 | 0.93779 |  | 1749.12 | 0.58561 |  | 663.393 | 0.77292 |
| **346.5** | 1.15067 |  | 1747.19 | 0.60285 |  | 661.464 | 0.77291 |
| 347.5 | 0.61466 |  | 1745.26 | 0.61879 |  | 659.536 | 0.76171 |
| 348 | 0.70797 |  | 1743.33 | 0.64007 |  | 549.613 | 0.85706 |
| **348.5** | 1.09918 |  | 1741.41 | 0.66040 |  | 541.899 | 0.89961 |
| 352.5 | 0.68673 |  | 1739.48 | 0.67448 |  | 539.971 | 0.91390 |
| 356.5 | 0.70754 |  | 1714.41 | 0.82848 |  | 538.042 | 0.92965 |
| **361.5** | 1.07075 |  | 1712.48 | 0.82484 |  | 536.114 | 0.94468 |
| **362** | 1.00013 |  | 1697.05 | 0.99738 |  | 534.185 | 0.95517 |
| 363 | 0.77221 |  | **1695.12** | 1.03424 |  | 532.257 | 0.96408 |
| 365 | 0.52438 |  | **1693.19** | 1.07046 |  | 491.759 | 0.96961 |
| 367 | 0.83056 |  | **1691.27** | 1.10929 |  | 489.831 | 0.98410 |
| 368 | 0.79205 |  | **1652.7** | 1.40688 |  | **416.549** | 1.36423 |
| 370 | 0.92622 |  | **1625.7** | 1.51786 |  | **401.121** | 1.50382 |
| 370.5 | 0.70267 |  | **1623.77** | 1.58807 |  |  |  |
| 372 | 0.80351 |  | **1621.84** | 1.64144 |  |  |  |
| 374 | 0.88001 |  | **1619.91** | 1.67505 |  |  |  |
| 374.5 | 0.70361 |  | 1583.27 | 0.82762 |  |  |  |
| 375.5 | 0.55675 |  | 1581.34 | 0.84377 |  |  |  |
| 376 | 0.83564 |  | 1579.41 | 0.86649 |  |  |  |
| 377.5 | 0.86448 |  | 1577.49 | 0.89861 |  |  |  |
| 378 | 0.68248 |  | 1575.56 | 0.93535 |  |  |  |
| **379.5** | 1.07454 |  | 1573.63 | 0.98019 |  |  |  |
| 381.5 | 0.73799 |  | 1554.34 | 0.99350 |  |  |  |
| **382.5** | 1.06365 |  | 1552.42 | 0.94257 |  |  |  |
| **386** | 1.01388 |  | 1550.49 | 0.88671 |  |  |  |
| 387.5 | 0.96847 |  | 1548.56 | 0.82883 |  |  |  |
| 389 | 0.85215 |  | 1544.7 | 0.73288 |  |  |  |
| 390 | 0.80460 |  | 1542.77 | 0.69484 |  |  |  |
| **391.5** | 1.10209 |  | 1540.84 | 0.66458 |  |  |  |
| 394.5 | 0.76808 |  | 1538.92 | 0.64260 |  |  |  |
| **396.5** | 1.06983 |  | 1536.99 | 0.62946 |  |  |  |

Notes: the variables with VIP scores greater than 1 are formatted in bold.

**Supplementary Table S4** VIPs of PLS-DA modeling on the epidermis of *W. cocos* with different collection regions.

| UV | |  | FTIR | |
| --- | --- | --- | --- | --- |
| Wavelength (nm) | VIP |  | Wavenumber (cm^-1^) | VIP |
| **200.5** | 1.76768 |  | 3999.64 | 0.78331 |
| **202** | 2.10912 |  | 3997.71 | 0.78226 |
| **203.5** | 1.81691 |  | 3995.78 | 0.78060 |
| **204** | 1.68635 |  | 3993.85 | 0.77797 |
| **204.5** | 1.63366 |  | 3991.93 | 0.77653 |
| **205** | 1.56290 |  | 3990 | 0.77362 |
| **206** | 1.30274 |  | 2983.34 | 0.92778 |
| **206.5** | 1.26295 |  | 2981.41 | 0.90871 |
| **207** | 1.29926 |  | 2979.48 | 0.90229 |
| **207.5** | 1.28443 |  | 2977.55 | 0.90696 |
| **208** | 1.14644 |  | 2975.62 | 0.91972 |
| **208.5** | 1.06703 |  | 2973.7 | 0.93647 |
| **209** | 1.05733 |  | 2971.77 | 0.95377 |
| 210 | 0.96479 |  | 2969.84 | 0.96971 |
| 210.5 | 0.96464 |  | 1795.4 | 0.44494 |
| 211 | 0.97817 |  | 1791.55 | 0.44440 |
| **212** | 1.01507 |  | 1789.62 | 0.44642 |
| 212.5 | 0.95783 |  | 1787.69 | 0.45001 |
| 213 | 0.97769 |  | 1785.76 | 0.45280 |
| 214 | 0.97441 |  | 1783.83 | 0.45441 |
| 214.5 | 0.99686 |  | 1781.9 | 0.45796 |
| 215 | 0.99685 |  | 1778.05 | 0.47772 |
| 216.5 | 0.96245 |  | 1741.41 | 0.87519 |
| 217.5 | 0.97613 |  | 1739.48 | 0.90871 |
| 220.5 | 0.97117 |  | 1737.55 | 0.94091 |
| 224.5 | 0.88241 |  | **1733.69** | 1.00829 |
| 225 | 0.83352 |  | **1731.76** | 1.04627 |
| 225.5 | 0.81267 |  | **1691.27** | 1.17796 |
| 226 | 0.81204 |  | **1689.34** | 1.16711 |
| 228.5 | 0.84482 |  | **1687.41** | 1.17177 |
| 230.5 | 0.97066 |  | **1685.48** | 1.18099 |
| 231 | 0.97968 |  | **1652.7** | 1.46303 |
| **232** | 1.20304 |  | **1650.77** | 1.45092 |
| **232.5** | 1.28507 |  | **1648.84** | 1.44567 |
| **234.5** | 1.17961 |  | **1646.91** | 1.44971 |
| **235** | 1.23424 |  | 1523.49 | 0.90060 |
| **235.5** | 1.41053 |  | 1521.56 | 0.91190 |
| **236** | 1.37428 |  | 1519.63 | 0.94996 |
| **236.5** | 1.14525 |  | **1517.7** | 1.00723 |
| **237** | 1.03198 |  | **1515.77** | 1.06851 |
| **238.5** | 1.36446 |  | **1455.99** | 1.13471 |
| **239.5** | 1.34173 |  | **1450.21** | 1.05560 |
| **240** | 1.83930 |  | **1353.78** | 1.03188 |
| **241.5** | 2.14727 |  | **1351.86** | 1.01682 |
| **242** | 2.58795 |  | 1349.93 | 0.99909 |
| **244.5** | 1.48322 |  | **1313.29** | 1.81760 |
| **245** | 1.60422 |  | **1311.36** | 1.63533 |
| **247** | 1.43931 |  | **1309.43** | 1.42014 |
| 257 | 0.73308 |  | **1209.15** | 1.12901 |
| 258 | 0.57358 |  | **1207.22** | 1.11360 |
| 258.5 | 0.54150 |  | **1205.29** | 1.10255 |
| 259 | 0.55892 |  | **1203.36** | 1.09647 |
| 259.5 | 0.52648 |  | **1184.08** | 1.16017 |
| 260 | 0.56034 |  | **1182.15** | 1.18430 |
| 260.5 | 0.54431 |  | **1180.22** | 1.21591 |
| 261 | 0.57624 |  | **1178.29** | 1.24946 |
| 261.5 | 0.56126 |  | **1176.36** | 1.28240 |
| 262 | 0.57466 |  | **1174.44** | 1.31249 |
| 262.5 | 0.57293 |  | **1149.37** | 1.29105 |
| 263 | 0.56194 |  | **1147.44** | 1.31588 |
| 263.5 | 0.57476 |  | **1145.51** | 1.31880 |
| 264 | 0.55582 |  | **1141.65** | 1.26830 |
| 264.5 | 0.56233 |  | **1108.87** | 1.32057 |
| 265 | 0.53335 |  | **1106.94** | 1.28779 |
| 265.5 | 0.56618 |  | **1105.01** | 1.26218 |
| 266 | 0.55466 |  | **1103.08** | 1.25081 |
| 278 | 0.64570 |  | **1101.15** | 1.25543 |
| 278.5 | 0.63100 |  | **1099.23** | 1.27856 |
| 279.5 | 0.64007 |  | **1068.37** | 1.59973 |
| 280 | 0.66896 |  | **1066.44** | 1.47949 |
| 280.5 | 0.65523 |  | **1064.51** | 1.38808 |
| 281 | 0.66208 |  | **1062.59** | 1.33674 |
| 281.5 | 0.67106 |  | **1045.23** | 1.32843 |
| 282 | 0.68254 |  | **1043.3** | 1.31626 |
| 282.5 | 0.67749 |  | **1041.37** | 1.32995 |
| 283 | 0.68087 |  | **1039.44** | 1.36421 |
| 283.5 | 0.68206 |  | **1037.52** | 1.40603 |
| 284 | 0.67542 |  | **1006.66** | 1.43388 |
| 284.5 | 0.68137 |  | **1004.73** | 1.38741 |
| 298 | 0.40263 |  | **1002.8** | 1.35320 |
| 299 | 0.41269 |  | **1000.87** | 1.35054 |
| 299.5 | 0.35266 |  | **998.946** | 1.39433 |
| 300 | 0.40873 |  | **997.017** | 1.48397 |
| 300.5 | 0.36172 |  | **632.537** | 1.19828 |
| 302 | 0.39024 |  | **630.609** | 1.19700 |
| 304 | 0.36119 |  | **609.396** | 1.17129 |
| 304.5 | 0.27023 |  | **607.467** | 1.17270 |
| 305 | 0.29794 |  | **605.539** | 1.17991 |
| 305.5 | 0.30585 |  | **603.61** | 1.18882 |
| 309 | 0.29706 |  | **528.4** | 1.28805 |
| 324 | 0.39906 |  | **526.471** | 1.23991 |
| 326.5 | 0.37659 |  | 499.473 | 0.83186 |
| 329 | 0.36295 |  | 497.544 | 0.84472 |
| 331.5 | 0.29189 |  | 495.616 | 0.87185 |
| 339 | 0.29326 |  | 493.688 | 0.91389 |
| 342.5 | 0.30656 |  | 491.759 | 0.97375 |
| 344 | 0.27047 |  | **489.831** | 1.04445 |
| 347.5 | 0.28482 |  | **474.403** | 1.40707 |
| 351 | 0.27506 |  | **472.474** | 1.42934 |
| 352 | 0.30675 |  | **470.546** | 1.44713 |
| 352.5 | 0.31378 |  | **468.617** | 1.45561 |
| 354 | 0.30294 |  | **466.689** | 1.45944 |
| 355 | 0.31392 |  | **449.333** | 1.18890 |
| 355.5 | 0.31402 |  | **447.404** | 1.14587 |
| 357.5 | 0.30580 |  | **445.476** | 1.11232 |
| 358.5 | 0.30567 |  | **443.547** | 1.08152 |
| 359 | 0.30775 |  | **441.619** | 1.06386 |
| 360.5 | 0.29944 |  | **439.69** | 1.07151 |
| 361.5 | 0.29977 |  | **437.762** | 1.09257 |
| 362.5 | 0.29262 |  | **435.833** | 1.11666 |
| 363.5 | 0.28898 |  | **412.692** | 1.24992 |
| 364 | 0.28508 |  | **410.763** | 1.27056 |
| 365 | 0.28756 |  | **408.835** | 1.29346 |
| 365.5 | 0.30769 |  | **406.906** | 1.35032 |
| 366.5 | 0.29356 |  |  |  |
| 367 | 0.31891 |  |  |  |
| 367.5 | 0.31243 |  |  |  |
| 368 | 0.32140 |  |  |  |
| 368.5 | 0.30784 |  |  |  |
| 370 | 0.30634 |  |  |  |
| 373.5 | 0.32116 |  |  |  |
| 379 | 0.33508 |  |  |  |
| 387 | 0.42087 |  |  |  |
| 388 | 0.42274 |  |  |  |
| 389.5 | 0.45379 |  |  |  |
| 390.5 | 0.50260 |  |  |  |
| 391.5 | 0.47844 |  |  |  |
| 392 | 0.46506 |  |  |  |
| 392.5 | 0.52384 |  |  |  |
| 393 | 0.49693 |  |  |  |
| 393.5 | 0.49684 |  |  |  |
| 394 | 0.51026 |  |  |  |
| 394.5 | 0.49989 |  |  |  |
| 395 | 0.53479 |  |  |  |
| 395.5 | 0.53912 |  |  |  |
| 396 | 0.54469 |  |  |  |
| 396.5 | 0.56238 |  |  |  |
| 397 | 0.55108 |  |  |  |
| 397.5 | 0.59315 |  |  |  |
| 398 | 0.56689 |  |  |  |
| 398.5 | 0.59021 |  |  |  |
| 399 | 0.59312 |  |  |  |
| 399.5 | 0.58235 |  |  |  |
| 400 | 0.59835 |  |  |  |

Notes: the variables with VIP scores greater than 1 are formatted in bold.

**Supplementary Table S5** VIPs of PLS-DA model according to different parts of *W. cocos* samples.

| UV | |  | FTIR | | | | | | | |
| --- | --- | --- | --- | --- | --- | --- | --- | --- | --- | --- |
| Wavelength (nm) | VIP |  | Wavenumber (cm^-1^) | VIP |  | Wavenumber (cm^-1^) | VIP |  | Wavenumber (cm^-1^) | VIP |
| **202** | 7.51119 |  | 3999.64 | 0.23745 |  | 1756.83 | 0.51988 |  | 1247.72 | 0.40681 |
| **202.5** | 7.47221 |  | 3997.71 | 0.23333 |  | 1752.98 | 0.57409 |  | 1245.79 | 0.40272 |
| **204** | 5.96246 |  | 3995.78 | 0.22922 |  | 1751.05 | 0.60388 |  | 1243.86 | 0.40550 |
| 214 | 0.82208 |  | 3993.85 | 0.22545 |  | 1747.19 | 0.66667 |  | 1241.93 | 0.41466 |
| 216 | 0.55175 |  | 3991.93 | 0.22184 |  | 1745.26 | 0.69957 |  | 1240 | 0.42912 |
| 216.5 | 0.49742 |  | 3990 | 0.21829 |  | 1743.33 | 0.73403 |  | 1141.65 | 0.47485 |
| 217 | 0.46425 |  | 3988.07 | 0.21461 |  | 1741.41 | 0.76877 |  | 1139.72 | 0.51395 |
| 220.5 | 0.41003 |  | 3986.14 | 0.21093 |  | 1737.55 | 0.83665 |  | 1137.8 | 0.56399 |
| 223 | 0.71738 |  | 3984.21 | 0.20732 |  | 1735.62 | 0.87086 |  | 1105.01 | 0.60568 |
| **226.5** | 1.18315 |  | 3982.28 | 0.20368 |  | 1733.69 | 0.90558 |  | 1101.15 | 0.64907 |
| **228** | 1.40183 |  | 3980.36 | 0.20006 |  | 1731.76 | 0.93925 |  | 1099.23 | 0.68221 |
| **232** | 2.08550 |  | 3978.43 | 0.19652 |  | 1729.83 | 0.97260 |  | 1097.3 | 0.72392 |
| **234** | 2.41592 |  | 3976.5 | 0.19301 |  | **1727.91** | 1.00649 |  | 1095.37 | 0.77359 |
| **234.5** | 2.47299 |  | 3972.64 | 0.18618 |  | **1710.55** | 1.27430 |  | 1093.44 | 0.83009 |
| **238** | 2.69790 |  | 3970.71 | 0.18293 |  | **1708.62** | 1.28214 |  | **1024.02** | 1.00330 |
| **239** | 2.81378 |  | 3968.78 | 0.17979 |  | **1706.69** | 1.27836 |  | 1022.09 | 0.94030 |
| **248.5** | 1.73904 |  | 3964.93 | 0.17381 |  | **1704.76** | 1.26097 |  | 1020.16 | 0.88275 |
| **250** | 1.60483 |  | 3963 | 0.17091 |  | **1702.84** | 1.23202 |  | 1018.23 | 0.82970 |
| **251** | 1.46799 |  | 3961.07 | 0.16808 |  | **1700.91** | 1.19320 |  | 1016.3 | 0.77889 |
| 263.5 | 0.48308 |  | 3959.14 | 0.16540 |  | **1698.98** | 1.14615 |  | 1014.37 | 0.72840 |
| 264 | 0.48654 |  | 3957.21 | 0.16283 |  | **1697.05** | 1.09518 |  | 997.017 | 0.81021 |
| 264.5 | 0.48646 |  | 3922.5 | 0.14522 |  | **1695.12** | 1.04334 |  | 995.089 | 0.95466 |
| 265.5 | 0.50384 |  | 3531.02 | 0.42123 |  | 1693.19 | 0.99393 |  | **993.16** | 1.10692 |
| 267.5 | 0.45111 |  | 3529.09 | 0.40301 |  | 1689.34 | 0.91655 |  | **991.232** | 1.25876 |
| 269.5 | 0.42362 |  | 3527.17 | 0.38733 |  | 1687.41 | 0.89146 |  | **989.303** | 1.40290 |
| 271.5 | 0.41813 |  | 3525.24 | 0.37440 |  | 1683.55 | 0.86966 |  | **987.375** | 1.53390 |
| 274.5 | 0.37065 |  | 3523.31 | 0.36430 |  | **1662.34** | 1.17602 |  | **985.447** | 1.64730 |
| 276.5 | 0.35954 |  | 3521.38 | 0.35710 |  | **1656.55** | 1.26584 |  | **983.518** | 1.73967 |
| 277.5 | 0.36781 |  | 3519.45 | 0.35280 |  | **1654.62** | 1.29349 |  | **964.233** | 1.58903 |
| 279 | 0.36726 |  | 3517.52 | 0.35141 |  | **1652.7** | 1.32209 |  | **962.305** | 1.51364 |
| 279.5 | 0.37006 |  | 3502.1 | 0.43235 |  | **1650.77** | 1.35073 |  | **960.376** | 1.43328 |
| 280.5 | 0.35159 |  | 3498.24 | 0.46940 |  | **1625.7** | 2.21027 |  | **958.448** | 1.34741 |
| 281.5 | 0.37445 |  | 3494.38 | 0.50824 |  | **1619.91** | 2.35980 |  | **956.52** | 1.25607 |
| 282.5 | 0.37885 |  | 3490.52 | 0.54721 |  | **1617.98** | 2.34468 |  | **954.591** | 1.16009 |
| 285 | 0.36495 |  | 3484.74 | 0.60418 |  | **1616.06** | 2.29731 |  | **952.663** | 1.05929 |
| 285.5 | 0.32675 |  | 3478.95 | 0.65763 |  | **1614.13** | 2.22116 |  | 917.95 | 0.48859 |
| 286.5 | 0.32232 |  | 3475.1 | 0.69174 |  | **1612.2** | 2.12187 |  | 916.022 | 0.52206 |
| 291 | 0.26029 |  | 3394.1 | 0.93698 |  | **1610.27** | 2.00620 |  | 914.093 | 0.55136 |
| 295 | 0.25879 |  | 3390.24 | 0.92528 |  | **1608.34** | 1.88282 |  | 912.165 | 0.57335 |
| 301 | 0.11679 |  | 3388.32 | 0.91941 |  | **1592.91** | 1.10154 |  | 910.236 | 0.58332 |
| 302.5 | 0.12321 |  | 3386.39 | 0.91380 |  | **1590.99** | 1.03355 |  | 908.308 | 0.57801 |
| 303 | 0.11748 |  | 3384.46 | 0.90817 |  | 1589.06 | 0.97378 |  | 906.379 | 0.55535 |
| 303.5 | 0.10970 |  | 3382.53 | 0.90242 |  | 1587.13 | 0.91906 |  | 904.451 | 0.51428 |
| 304.5 | 0.13349 |  | 3380.6 | 0.89693 |  | 1585.2 | 0.86642 |  | **808.028** | 1.14401 |
| 309 | 0.12276 |  | 3378.67 | 0.89194 |  | 1583.27 | 0.81656 |  | **806.099** | 1.21470 |
| 317 | 0.12001 |  | 3376.75 | 0.88713 |  | 1581.34 | 0.76913 |  | **804.171** | 1.28315 |
| 318 | 0.16166 |  | 3374.82 | 0.88201 |  | 1579.41 | 0.72271 |  | **802.242** | 1.34139 |
| 325.5 | 0.15101 |  | 3372.89 | 0.87632 |  | 1577.49 | 0.67846 |  | **800.314** | 1.38144 |
| 326.5 | 0.15099 |  | 3370.96 | 0.87016 |  | 1575.56 | 0.63610 |  | **798.385** | 1.39938 |
| 328.5 | 0.17059 |  | 3369.03 | 0.86351 |  | 1573.63 | 0.59619 |  | **796.457** | 1.39784 |
| 329 | 0.13189 |  | 3367.1 | 0.85641 |  | 1571.7 | 0.56125 |  | **781.029** | 1.49452 |
| 329.5 | 0.12075 |  | 3365.17 | 0.84888 |  | 1569.77 | 0.52832 |  | **779.101** | 1.47153 |
| 330.5 | 0.15431 |  | 3363.25 | 0.84128 |  | 1536.99 | 0.20131 |  | **777.172** | 1.42192 |
| 331 | 0.15625 |  | 2960.2 | 0.37286 |  | 1535.06 | 0.19665 |  | **775.244** | 1.34512 |
| 331.5 | 0.13661 |  | 2958.27 | 0.34910 |  | 1533.13 | 0.19424 |  | **773.315** | 1.24424 |
| 332 | 0.17063 |  | 2956.34 | 0.32536 |  | 1531.2 | 0.19352 |  | 759.816 | 0.68093 |
| 333 | 0.14446 |  | 2954.41 | 0.30398 |  | 1529.27 | 0.19432 |  | 757.887 | 0.63958 |
| 334 | 0.15457 |  | 2950.55 | 0.27749 |  | 1527.35 | 0.19708 |  | 755.959 | 0.60002 |
| 335.5 | 0.11609 |  | 2832.92 | 0.60676 |  | 1432.85 | 0.97008 |  | 754.031 | 0.56216 |
| 336 | 0.15298 |  | 2829.06 | 0.58140 |  | 1430.92 | 0.96293 |  | 696.177 | 0.31688 |
| 337.5 | 0.17466 |  | 2310.3 | 0.28745 |  | 1428.99 | 0.95394 |  | 694.248 | 0.31251 |
| 338.5 | 0.18208 |  | 2306.45 | 0.28524 |  | 1427.07 | 0.94432 |  | 692.32 | 0.30803 |
| 339.5 | 0.15112 |  | 2304.52 | 0.28393 |  | 1425.14 | 0.93391 |  | 690.391 | 0.30499 |
| 344 | 0.11144 |  | 2302.59 | 0.28234 |  | 1423.21 | 0.92187 |  | 688.463 | 0.30688 |
| 345.5 | 0.14992 |  | 2300.66 | 0.28041 |  | 1421.28 | 0.90742 |  | 686.534 | 0.31679 |
| 348 | 0.14098 |  | 2298.73 | 0.27816 |  | 1419.35 | 0.89144 |  | 684.606 | 0.33479 |
| 351.5 | 0.16554 |  | 2296.8 | 0.27555 |  | 1417.42 | 0.87441 |  | 682.677 | 0.35727 |
| 352.5 | 0.18710 |  | 2067.32 | 0.13868 |  | 1415.49 | 0.85671 |  | 518.758 | 0.65119 |
| 353 | 0.16411 |  | 2063.46 | 0.13981 |  | 1413.57 | 0.83966 |  | 516.829 | 0.64353 |
| 354.5 | 0.17979 |  | 2061.53 | 0.14046 |  | 1411.64 | 0.82385 |  | 514.901 | 0.62865 |
| 357 | 0.16566 |  | 2059.6 | 0.14089 |  | 1409.71 | 0.80908 |  | 512.972 | 0.60609 |
| 357.5 | 0.16973 |  | 2053.82 | 0.14319 |  | 1407.78 | 0.79600 |  | 511.044 | 0.57833 |
| 358.5 | 0.17619 |  | 2051.89 | 0.14420 |  | 1405.85 | 0.78566 |  | 509.115 | 0.54955 |
| 359 | 0.14090 |  | 2048.03 | 0.14650 |  | 1403.92 | 0.77831 |  | 507.187 | 0.52287 |
| 361 | 0.19714 |  | 2046.1 | 0.14785 |  | 1361.5 | 0.74793 |  | 487.902 | 0.67608 |
| 362 | 0.18699 |  | 2044.17 | 0.14969 |  | 1359.57 | 0.71369 |  | 485.974 | 0.76207 |
| 363.5 | 0.20179 |  | 2042.25 | 0.15151 |  | 1357.64 | 0.68040 |  | 484.045 | 0.86063 |
| 364.5 | 0.21535 |  | 2040.32 | 0.15299 |  | 1355.71 | 0.64956 |  | 482.117 | 0.96746 |
| 366 | 0.20050 |  | 2038.39 | 0.15465 |  | 1353.78 | 0.62081 |  | **480.188** | 1.07713 |
| 369.5 | 0.18262 |  | 2036.46 | 0.15675 |  | 1351.86 | 0.59270 |  | **468.617** | 1.49199 |
| 370.5 | 0.16678 |  | 2034.53 | 0.15896 |  | 1349.93 | 0.56361 |  | **466.689** | 1.49864 |
| 372 | 0.14279 |  | 2030.68 | 0.16339 |  | 1348 | 0.53201 |  | **464.76** | 1.48658 |
| 374 | 0.14927 |  | 2028.75 | 0.16556 |  | 1346.07 | 0.49732 |  | **462.832** | 1.45673 |
| 377 | 0.15228 |  | 2026.82 | 0.16783 |  | 1344.14 | 0.46079 |  | **460.904** | 1.41054 |
| 378 | 0.16100 |  | 2024.89 | 0.17011 |  | 1342.21 | 0.42292 |  | **458.975** | 1.35161 |
| 378.5 | 0.16591 |  | 2022.96 | 0.17215 |  | 1340.28 | 0.38469 |  | **457.047** | 1.28360 |
| 379.5 | 0.16230 |  | 2019.1 | 0.17638 |  | 1338.36 | 0.34943 |  | **455.118** | 1.20968 |
| 380.5 | 0.21861 |  | 2017.18 | 0.17846 |  | 1336.43 | 0.32181 |  | **453.19** | 1.13123 |
| 381.5 | 0.17727 |  | 2015.25 | 0.18008 |  | 1334.5 | 0.30497 |  | **451.261** | 1.04927 |
| 383 | 0.21322 |  | 2009.46 | 0.18570 |  | 1332.57 | 0.29971 |  | 443.547 | 0.76575 |
| 384 | 0.17478 |  | 2007.53 | 0.18736 |  | 1319.07 | 0.50765 |  | 441.619 | 0.71709 |
| 385 | 0.14710 |  | 2003.68 | 0.18974 |  | 1317.14 | 0.49652 |  | 437.762 | 0.64084 |
| 385.5 | 0.20850 |  | 1999.82 | 0.19193 |  | 1315.21 | 0.46553 |  | 435.833 | 0.61155 |
| 390.5 | 0.14616 |  | 1992.11 | 0.19369 |  | 1313.29 | 0.41316 |  | 433.905 | 0.58491 |
| 392 | 0.17006 |  | 1984.39 | 0.19258 |  | 1311.36 | 0.35683 |  | 431.977 | 0.56166 |
| 392.5 | 0.20488 |  | 1982.46 | 0.19226 |  | 1309.43 | 0.30119 |  | 430.048 | 0.54148 |
| 393 | 0.14961 |  | 1976.68 | 0.19158 |  | 1295.93 | 0.24930 |  | 410.763 | 0.44778 |
| 394.5 | 0.18285 |  | 1974.75 | 0.19190 |  | 1294 | 0.25920 |  | 408.835 | 0.46307 |
| 397 | 0.17748 |  | 1963.18 | 0.19360 |  | 1292.07 | 0.26783 |  | 406.906 | 0.47409 |
| 397.5 | 0.21890 |  | 1951.61 | 0.19917 |  | 1290.14 | 0.27401 |  | 403.05 | 0.45446 |
|  |  |  | 1774.19 | 0.34556 |  | 1288.22 | 0.27695 |  | 401.121 | 0.48765 |
|  |  |  | 1772.26 | 0.36179 |  | 1286.29 | 0.27650 |  |  |  |
|  |  |  | 1766.48 | 0.41367 |  | 1249.65 | 0.41778 |  |  |  |

Notes: the variables with VIP scores greater than 1 are formatted in bold.

**Supplementary Table S6** Voucher specimens deposition numbers of *W. cocos*.

| No | Voucher specimens No |  | No | Voucher specimens No |
| --- | --- | --- | --- | --- |
| 1-5 | YW-FLH-YX 1-5 |  | 176-180 | YW-FLP-YX 1-5 |
| 6-10 | YW-FLH-YX 6-10 |  | 181-185 | YW-FLP-YX 6-10 |
| 11-15 | YW-FLH-YX 11-15 |  | 186-190 | YW-FLP-YX 11-15 |
| 16-20 | YW-FLH-YX 16-20 |  | 191-195 | YW-FLP-YX 16-20 |
| 21-25 | YW-FLH-YX 21-25 |  | 196-200 | YW-FLP-YX 21-25 |
| 26-30 | YW-FLH-YX 26-30 |  | 201-205 | YW-FLP-YX 26-30 |
| 31-35 | YW-FLH-YX 31-35 |  | 206-210 | YW-FLP-YX 31-35 |
| 36-40 | YW-FLH-YX 36-40 |  | 211-215 | YW-FLP-YX 36-40 |
| 41-45 | YW-FLH-YX 41-45 |  | 216-220 | YW-FLP-YX 41-45 |
| 46-50 | YW-FLH-WS 46-50 |  | 221-225 | YW-FLP-WS 46-50 |
| 51-55 | YW-FLH-WS 51-55 |  | 226-230 | YW-FLP-WS 51-55 |
| 56-60 | YW-FLH-WS 56-60 |  | 231-235 | YW-FLP-WS 56-60 |
| 61-65 | YW-FLH-HH 61-65 |  | 236-240 | YW-FLP-HH 61-65 |
| 66-70 | YW-FLH-HH 66-70 |  | 241-245 | YW-FLP-HH 66-70 |
| 71-75 | YW-FLH-HH 71-75 |  | 246-250 | YW-FLP-HH 71-75 |
| 76-80 | YW-FLH-HH 76-80 |  | 251-255 | YW-FLP-HH 76-80 |
| 81-85 | YW-FLH-PE 81-85 |  | 256-260 | YW-FLP-PE 81-85 |
| 86-90 | YW-FLH-PE 86-90 |  | 261-265 | YW-FLP-PE 86-90 |
| 91-95 | YW-FLH-PE 91-95 |  | 266-270 | YW-FLP-PE 91-95 |
| 96-100 | YW-FLH-CX 96-100 |  | 271-275 | YW-FLP-CX 96-100 |
| 101-105 | YW-FLH-CX 101-105 |  | 276-280 | YW-FLP-CX 101-105 |
| 106-110 | YW-FLH-CX 106-110 |  | 281-285 | YW-FLP-CX 106-110 |
| 111-115 | YW-FLH-CX 111-115 |  | 286-290 | YW-FLP-CX 111-115 |
| 116-120 | YW-FLH-CX 116-120 |  | 291-295 | YW-FLP-CX 116-120 |
| 121-125 | YW-FLH-NJ 121-125 |  | 296-300 | YW-FLP-NJ 121-125 |
| 126-130 | YW-FLH-NJ 126-130 |  | 301-305 | YW-FLP-NJ 126-130 |
| 131-135 | YW-FLH-BS 131-135 |  | 306-310 | YW-FLP-BS 131-135 |
| 136-140 | YW-FLH-BS 136-140 |  | 311-315 | YW-FLP-BS 136-140 |
| 141-145 | YW-FLH-LC 141-145 |  | 316-320 | YW-FLP-LC 141-145 |
| 146-150 | YW-FLH-LC 146-150 |  | 321-325 | YW-FLP-LC 146-150 |
| 151-155 | YW-FLH-LC 151-155 |  | 326-330 | YW-FLP-LC 151-155 |
| 156-160 | YW-FLH-PE-C 156-160 |  | 331-335 | YW-FLP-PE-C 156-160 |
| 161-165 | YW-FLH-PE-C 161-165 |  | 336-340 | YW-FLP-PE-C 161-165 |
| 166-170 | YW-FLH-PE-C 166-170 |  | 341-345 | YW-FLP-PE-C 166-170 |
| 171-175 | YW-FLH-PE-C 171-175 |  | 346-350 | YW-FLP-PE-C 171-175 |
